# Effect of Disorder on the Emission Spectra of Er^3+^, Tm^3+^, and Yb^3+^-Doped β‑NaYF_4_: Quantum Chemical and Experimental Results

**DOI:** 10.1021/acs.jpcc.5c06209

**Published:** 2025-10-30

**Authors:** Chris Steve Conrad, Stefan Behnle, Eva Hemmer, Reinhold F. Fink

**Affiliations:** † Institute of Physical and Theoretical Chemistry, 9188 University of Tübingen, Tübingen, Baden-Württemberg 72076, Germany; ‡ Department of Chemistry and Biomolecular Sciences, 6363 University of Ottawa, Ottawa, Ontario K1N 6N5, Canada

## Abstract

Exact optical characteristics of the trivalent lanthanide ions (Ln^3+^) are crucially determined by their nearest neighbors and in turn govern the quantum efficiency of Ln^3+^-doped upconverting nanoparticles (UCNPs). Their often extremely low quantum efficiency can be increased considerably by doping Ln^3+^ into disordered host lattices. In these lattices, each Ln^3+^ experiences a slightly different low-symmetry environment, which leads to small variations in its (optical) properties. To this end, we predict crystal field energy levels and oscillator strengths of Er^3+^, Tm^3+^, and Yb^3+^ doped into disordered hexagonal (β-)­NaYF_4_ and ordered LiYF_4_. In addition, theoretical photoluminescence spectra for β-NaYF_4_:Er^3+^ were determined. The results were obtained using a wave function-based ab initio computational approach and an embedded cluster model. The disorder of β-NaYF_4_:Ln^3+^ was accounted for through weighted averaging, in which stochastic considerations and Boltzmann distributions for several local configurations were included. Comparison to experimental and semiempirical data showed particularly good agreement for both the ordered and disordered material. The disordered lattice significantly shifted the crystal field energy levels and changed the oscillator strengths of the respective Ln^3+^ transitions. Importantly, the computed photoluminescence spectra showed the best agreement with experimental spectra when including the predicted results of all disordered configurations individually. This reveals that the observed energetic splitting and transition rates of the excited Ln^3+^ dopants can only be accurately described if the disorder of the β-NaYF_4_ crystal is properly considered. The proposed quantum chemical protocol paves the way for the future simulation of photon upconversion processes in UCNPs.

## Graphical abstract

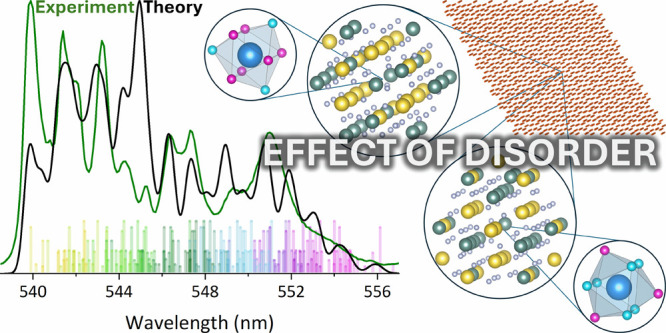

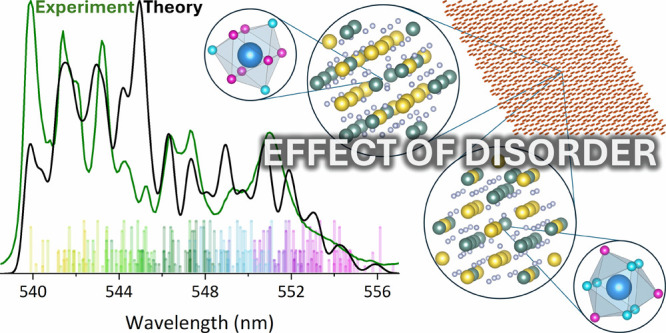

## Introduction

In recent years, growing attention has been dedicated to photon upconversion and how employing upconverting nanoparticles (UCNPs) might help tackling current societal challenges such as treating cancer, detecting potential heart failure, in vivo temperature measurements and manipulations, ensuring food safety, or monitoring aquatic organisms.^1–7^ In fact, the underlying fundamentals of the upconversion process have been well-studied in more than 50 years since the phenomenon has been first described.^8,9^ Most upconverting systems operate by the energy transfer upconversion (ETU) mechanism, which can be split into four separate steps: (1) A photon is absorbed by an ion, and the ion uses the photon’s energy for reaching an exited state. (2) The energy is nonradiatively transferred to a second ion. As a result, the second ion is excited while the first ion relaxes back to the ground state. (3) Another photon is absorbed by the first or another ion, and the energy is again transferred to the second ion. Since the second ion is already in an exited state, it uses the transferred energy to reach an even higher exited state. (4) If the second ion relaxes back to the ground state radiatively, a photon with more energy than each of the initially absorbed photons is emitted, finalizing the upconversion process.^10^

Efficient upconverters are several of the trivalent lanthanide ions (Ln^3+^) doped into inorganic host crystals, e.g., cubic- (α) and hexagonal- (β) phase NaYF_4_ and NaGdF_4_ or LiYF_4_ doped with Er^3+^ or Tm^3+^ and Yb^3+^ as a codopant.^11–13^ The 4f–4f transitions of the Ln^3+^ in these matrices combine long lifetimes of the excited states with still meaningful transition rates between these states.^14,15^ Both properties are needed for an efficient upconversion process. The transition rates between the energy levels can be quantified by the respective oscillator strengths. Because of Laporte’s rule, these oscillator strengths are near to zero for lanthanides in a centrosymmetric environment. However, once the Ln^3+^ are subjected to a noncentrosymmetric surrounding by a host crystal, the previously degenerate states (multiplets) split into crystal field energy levels called Stark levels. Due to the reduced symmetry, larger transition rates can be observed between these states. Still, the quantum yield remains low for UCNPs, with values typically being in the range of less than 1%,^16,17^ slowing UCNPs on their way to real-life applicability, although progress has been made.^18–20^

To overcome this challenge, a variety of strategies has been developed over the years to boost the upconversion quantum yield of nanoparticles.^20–23^ Notably, record emissions—recently up to 13%—were commonly observed for dopant–host systems with a disordered host lattice.^18,24–27^ Possible sources of disorder within a crystal lattice are sites that are occupied only partially or by different ions. The higher quantum yield in disordered crystals has been associated with the Ln^3+^ ions being doped into low-symmetry sites, which results in higher transition intensities. In turn, this results in a more efficient upconversion process assuming all other parameters are kept the same. Moreover, in a disordered host, the Ln^3+^ are doped into sites that all differ marginally, but importantly not negligibly, from each other.^28,29^ Therefore, each Ln^3+^ has energy levels with slightly different positions, and the transitions between these energy levels have different oscillator strengths. These nuanced changes can potentially be beneficial in the energy transfer processes as needed for ETU and thereby also aid in increasing the quantum yield of UCNPs.

In an ETU process that involves two different types of ions, e.g., Er^3+^ and Yb^3+^ or Tm^3+^ and Yb^3+^, the energy levels of the donor and acceptor ions usually do not align perfectly causing an energy mismatch.^30^ This energy mismatch and the oscillator strengths of the involved transitions crucially determine the efficiency of the energy transfer process.^31^ To elaborate on this further, in any dopant–host system, the donor (i.e., Yb^3+^) has a certain number of acceptor ions (i.e., Er^3+^ or Tm^3+^) in its immediate surrounding. In an ordered host crystal, all these acceptors (and donors) have the same properties. In our previous work, we implied that in the disordered β-NaYF_4_ crystal, ions will be doped into a variety of low-symmetry sites, all of which have slightly different environments.^29^ Therefore, all of the dopants should exhibit slightly varying properties. If any combination of a donor and an acceptor results in a smaller energy mismatch coupled with higher oscillator strengths, the efficiency of the energy transfer process and thereby the quantum yield of the system should increase compared to that of an ordered system.

Shyichuk et al. previously modeled ETU and the corresponding upconversion processes for Er^3+^ and Yb^3+^ within an ordered host crystal (YVO_4_), using a semiempirical approach.^31^ Conversely, to the best of our knowledge, disordered hosts have not been addressed to date. A comparative study where similar ordered and disordered host materials are modelled on the same footing could provide evidence that disordered host crystals are especially beneficial for ETU processes. Moreover, such modeling has the potential to foster the discovery of even better host materials or dopant–host combinations. However, because of the enormous number of configurations observed in disordered crystals, the semiempirical approach of Shyichuk et al. is not suitable for this endeavor. Consequently, a different computational approach must be followed, where ab initio strategies stand out as a suitable alternative. More precisely, we propose here an ab initio computational model to probe properties like crystal field energy levels and oscillator strengths of three different Ln^3+^ (i.e., Er^3+^, Tm^3+^, and Yb^3+^) for both an ordered (LiYF_4_) and a disordered (β-NaYF_4_) host crystal.

In the following, first, the structural properties of the ordered and disordered host materials and its dopants will be summarized based on our previous investigation.^29^ The sources of disorder in β-NaYF_4_ will also be highlighted, followed by the setup of the ab initio calculations for both the ordered and the disordered systems. Having a suitable model on hand, the energy levels and oscillator strengths of the Ln^3+^ under consideration could be determined. Importantly, the modeled data, i.e., energy levels, oscillator strengths, and photoluminescence spectra, showed good agreement with data taken from the literature as well as experimentally obtained emission spectra from UCNPs synthesized in-house.^32–40^ Our calculations also provide evidence that a single configuration of atomic positions is insufficient to predict the optical properties of a disordered structure. Instead, at least a representative ensemble of possible configurations must be included to obtain meaningful insights into such materials. These insights have the potential to contribute to the discovery of novel dopant–host combinations for UCNPs, with highly efficient ETU and increased quantum yield, possibly fostering their real-life application.

## Theoretical Setup

### β-NaYF_4_ Disordered Crystal Structure

The unit cell of β-NaYF_4_ is shown in Figure 1A. Assuming the *P*6̅ space group for its lattice, it contains two disordered sites.^41^ One site, at Wyckoff position 1f, is occupied by either Na^+^ or Y^3+^, with equal probabilities for both ions. The other disordered site consists of two connected sites, both at Wyckoff position 2h. In each unit cell, exactly one of the two 2h sites is occupied by Na^+^, with equal probability. The β-NaYF_4_ crystal structure is therefore highly irregular even without accounting for additional defects such as vacancies, dopants, or interstitial atoms.^42,43^ Consequently, from a computational perspective, the accurate description of this structure is challenging but has been addressed before.^44–47^ In our previously reported approach,^29^ we proposed the creation of a 2 × 2 × 4 supercell of β-NaYF_4_ and alteration of the distribution of Na^+^ and Y^3+^ at the two disordered sites around the two potential Ln^3+^ doping sites (at the Wyckoff positions 1f and 1a). Hereby, the overall composition of the supercell was kept constant, while 16 supercells with different configurations were created to represent the disordered crystal. These 16 configurations were subsequently doped with either Er^3+^, Tm^3+^, or Yb^3+^ and then structurally optimized employing a density functional theory (DFT) with periodic boundary conditions ansatz. One of these doped supercells is shown in Figure 1B. More details on the computational ansatz can be found in the original publication.^29^ The different arrangements of Na^+^ and Y^3+^ in each supercell induced slight rearrangements of all other ions in the immediate surroundings, including the anions (F^–^) next to the Ln^3+^ doping sites. Therefore, each supercell exhibited slightly varying structural properties after the optimization, and nuanced changes were observed in the environments of the available doping sites.^29^ From these supercells, cluster embeddings were generated to probe the electronic properties of the dopants.

**Figure 1:**
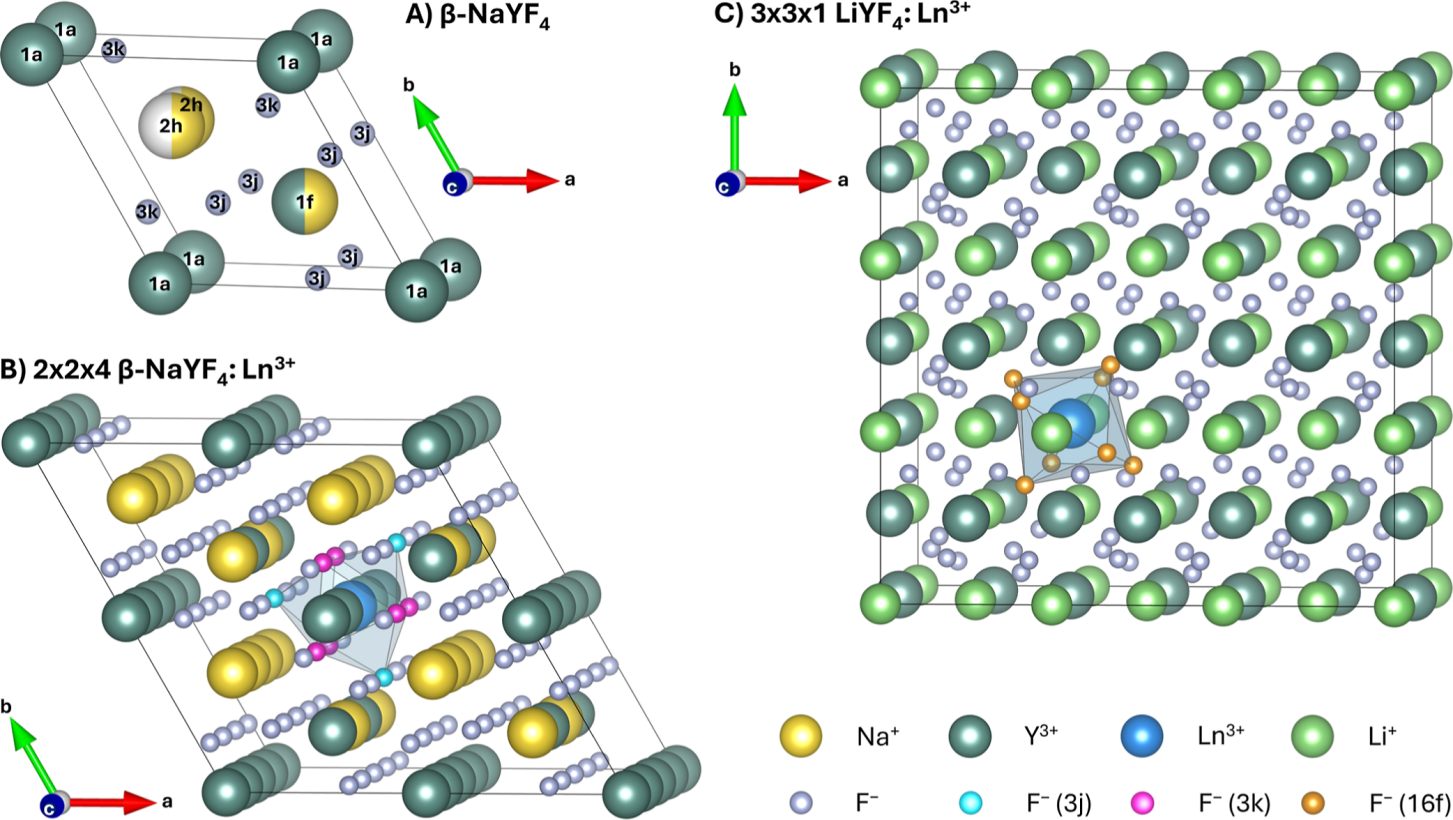
Unit cell and Ln^3+^-doped supercells of β-NaYF_4_ and LiYF_4_. (A) Unit cell of β-NaYF_4_ (space group *P*6̅) with the composition Na_1.5_Y_1.5_F_6_ and labeling of the Wyckoff sites. Yellow spheres represent Na^+^, dark green spheres Y^3+^, small gray spheres F^–^, white spheres are unoccupied sites, and half spheres indicate a 50% occupation. (B) 2 × 2 × 4 supercell of β-NaYF_4_:Ln^3+^ with the composition Na_24_Y_23_Ln_1_F_96_. In addition to the color code used in (A), blue spheres represent Ln^3+^, and small cyan and pink spheres stand for the nine F^–^ at the two different Wyckoff positions closest to the Ln^3+^ doping site. For better visualization, a polyhedron (tricapped trigonal prism) was drawn around the Ln^3+^ doping site, connecting the F^–^ at the edges of the polyhedron. The Ln^3+^ ion is doped into one of two possible sites (Wyckoff position 1a, alternatively 1f). (C) 3 × 3 × 1 supercell of LiYF_4_:Ln^3+^ with the composition Li_36_Y_35_Ln_1_F_144_. In addition to the color code used in (A), light green spheres represent Li^+^, and small orange spheres stand for the eight F^–^ closest to the Ln^3+^ doping site. The included polyhedron (distorted square antiprism) connects the F^–^ neighboring the central Ln^3+^ doping site. The space group of the unit cell used to construct the supercell is *I*4_1_/*a* (tetragonal).

### Cluster Embedding

#### β-NaYF_4_:Ln^3+^

Initially, 16 individual supercells for each of the three Ln^3+^ considered herein (i.e., Er^3+^, Tm^3+^, and Yb^3+^, 48 in total) were available for further investigation. Of the 16 supercells for each Ln^3+^, the 14 with the lowest energy were considered for the current work, excluding two supercells for each Ln^3+^. Thereby, the total number of structures considered for β-NaYF_4_:Ln^3+^ was reduced to 42. The unfavorable structural arrangements for the two omitted configurations resulted in significantly increased energies of the supercells, making them unlikely to be found in real crystals. Details on the exact arrangements of the Na^+^ and Y^3+^ around the respective doping sites can be found in our previous work.^29^

An ionic cluster embedding scheme based on these supercells was created following the procedure reviewed by Staemmler,^48^ including the adjustments introduced by Aravena et al. and Atanasov et al.^49,50^ For each supercell, an embedded cluster model was designed as described in the following. The embedding scheme for one of the clusters is depicted in Figure 2. This cluster is derived from supercell 10 of our previous work and is used most often when β-NaYF_4_ is treated as an ordered crystal.^29,51–53^ The numbering for the different supercells is explained in detail below. For this particular structure, Na^+^ and Y^3+^ at the 1f sites form regularly alternating columns (Figure 1B). For further illustrations of the supercells with different Na^+^-Y^3+^ arrangements, the interested reader is referred to our previous publication and its Supporting Information.^29^

**Figure 2:**
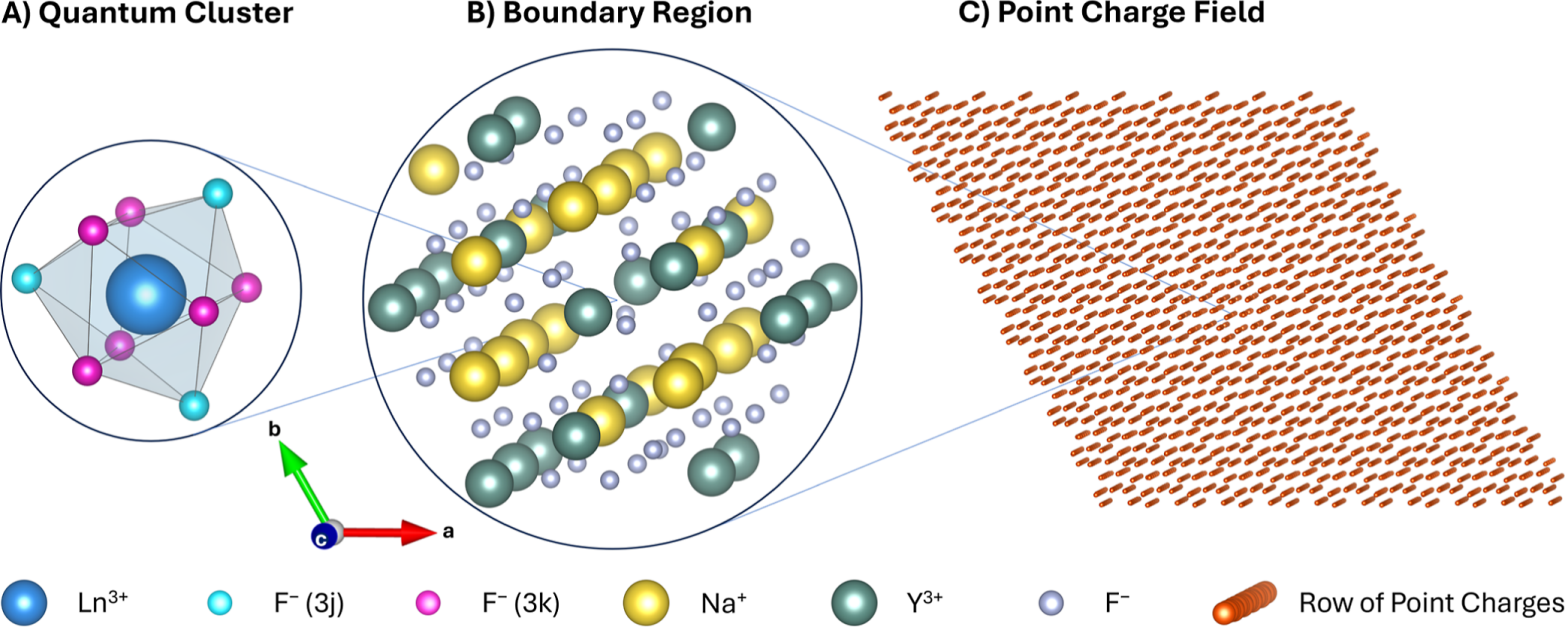
Ionic cluster embedding for one cluster of β-NaYF_4_:Ln^3+^. (A) LnF_9_ quantum cluster. (B) Na_19_Y_22_F_62_ boundary region. (C) Field with 17,886 point charges.

#### Quantum Cluster

For creating each cluster, the central Ln^3+^ ion and its closest neighbors (nine F^–^ for β-NaYF_4_) were cut from the supercell (Figure 1B). These ions formed the quantum cluster, Figure 2A, in which all interactions between all electrons of all ions were calculated explicitly at a quantum-mechanical level (see the section Computational Details). Although the composition of the quantum cluster is the same for all 42 clusters of β-NaYF_4_ (one Ln^3+^ plus nine F^–^), the positions of the F^–^ ions within each cluster vary slightly. This is predominantly due to the changing distribution of Na^+^ and Y^3+^ being directly adjacent.

#### Boundary Region

The ions in the supercell adjacent to the Ln^3+^ and F^–^ of the core quantum cluster are included into the setup by adding them to the boundary region (Figure 2B). More precisely, the boundary region forms an intermediate layer between the quantum cluster and the point charge field (Figure 2C). Ions in the boundary region are approximated by a point charge plus an effective core potential (ECP). The term we prefer to use for these is therefore capped point charges.^54^ The reason for equipping point charges closest to the quantum clusters with ECPs is to accurately mimic the Pauli repulsion between the electrons of the F^–^ in the quantum cluster and the core electrons of the surrounding ions. This addition prevents unphysical “leaking” of electrons into the boundary region. Also, by representing an ion not only by its charge but also by a potential that mimics the electronic structure of the original ion at least to a certain degree, the electrons in the quantum cluster are surrounded by an environment that better reflects the conditions in a real crystal. This allows for minimizing the size of the quantum cluster, which drastically reduces computational effort, while only marginally influencing the obtained results.^49,54^

The size of the boundary region for all clusters was chosen such that approximately 23 Y^3+^ were included as capped point charges. The exact number depended on the distance of each ion to the central Ln^3+^ and the structural configuration. 23 Y^3+^ plus 1 Ln^3+^ was also the same distribution as found in the supercells from which the embedding was created.^29^ The ratio of one Ln^3+^ in the center to the number of Y^3+^ in the boundary region corresponded to a doping concentration of 4–5 mol % for the quantum cluster and boundary region. Overall, the boundary region typically consisted of 110 capped point charges arranged in about two double layers around the quantum cluster. Figure 3A depicts the quantum cluster and the first layer of the boundary region for the cluster with the highest weighting factor (explained below). In Figure 3B, the same information is provided for the cluster commonly used for β-NaYF_4_:Ln^3+^ in other works.

**Figure 3:**
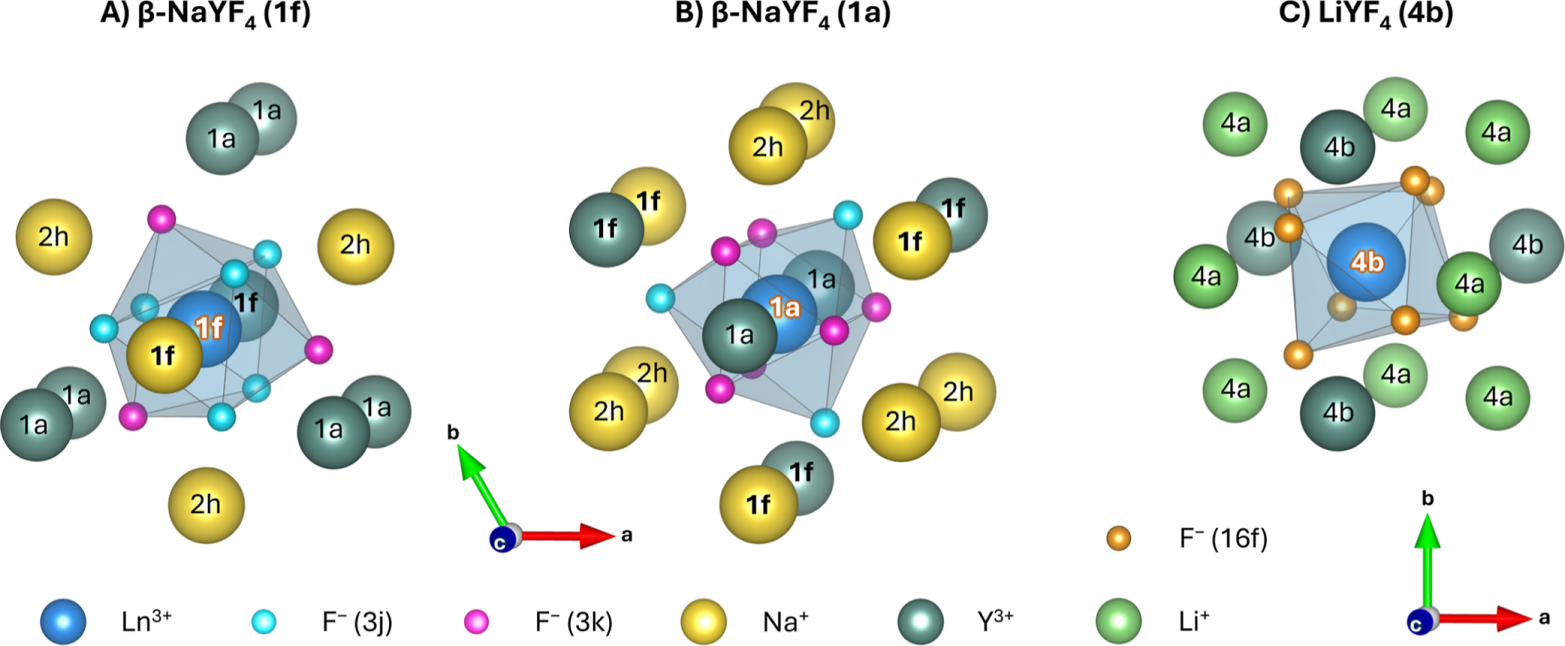
Examples for quantum clusters and first layer of the boundary regions for β-NaYF_4_:Ln^3+^ (A,B) and LiYF_4_:Ln^3+^ (C). (A) Two disordered 1f sites (set in bold) are part of the first anionic coordination sphere, and one of the two sites is occupied by Y^3+^. (B) Six 1f sites are included, and three of these are occupied by Y^3+^. Variations of the clusters are possible not only by changing the ratio of Na^+^ to Y^3+^ at the 1f sites but also by arranging them differently at the available sites. Through the varied distribution of Na^+^ and Y^3+^ at the disordered sites and the slightly differing radii of these ions, the F^–^ in the quantum cluster are located at marginally different positions, thereby changing the electronic properties of the central Ln^3+^. Na^+^ ions at the 2h sites are also subject to disorder, having a similar influence. (C) No disordered sites are present in the crystal structure of LiYF_4_:Ln^3+^; exactly one anionic coordination sphere exists.

#### Point Charge Field

The third part of the embedding consisted of pure ionic point charges, their positions being iteratively added as additional layers around the boundary region (Figure 2C). These point charges were incorporated to model the electrostatic potential (long-range Coulomb effects) of the host crystal. Typically, up to 18,000 charges were added for β-NaYF_4_:Ln^3+^. The size of the created clusters corresponded to nanoparticles of approximately 5 nm in diameter. The charges of the point charges were slightly adjusted to reproduce the correct electrostatic potential of the crystal at the embedding site.^55^

With these three layers discussed above, the surroundings of the Ln^3+^ dopant were set up to model the real environment experienced by the central ion as accurately as possible. However, this setup also assumed that the particle is defect-free and a single Ln^3+^ is doped almost exactly into the cluster’s center.

#### LiYF_4_:Ln^3+^

A similar procedure for creating the ionic embedding was applied for LiYF_4_:Ln^3+^. It differed from that of β-NaYF_4_:Ln^3+^ in the number of F^–^ in the quantum cluster (eight for LiYF_4_:Ln^3+^) and through the associated structural changes. Additionally, the boundary region consisted of Li^+^, Y^3+^, and F^–^, and the point charge field had up to 27,000 charges. Also, since the unit cell of LiYF_4_ is completely ordered, a single configuration sufficed to describe its lattice. Therefore, only three cluster embeddings were created for this crystal, one for each of the Ln^3+^ under consideration, i.e., Er^3+^, Tm^3+^, and Yb^3+^. The supercell for LiYF_4_:Ln^3+^ is shown in Figure 1C.

Two additional calculations were conducted for LiYF_4_ doped with either Er^3+^ or Yb^3+^. Herein, the first layer of the boundary region (Li_8_Y_4_, Figure 3C) was added to the quantum cluster to test the size convergence of our model. The resulting energy levels shifted on average by less than 0.1%, while the computational demand increased significantly by more than 1 order of magnitude. No further structures with the increased size of the quantum cluster were therefore considered. The output files containing the respective data sets for these and for all other calculations mentioned in this study are provided via a research data repository (RDR), to which a link is provided in the section Associated Content.

### Computational Details

The previously optimized geometries were converted from the CP2K output into CIF files with a Python script and the VESTA crystal visualization program by Momma and Izumi.^56^ The VESTA software package was also used for drawing all of the shown crystal structures and cluster embeddings. The thereby obtained CIF files were utilized for the creation of the ionic cluster embedding (i.e., quantum cluster, boundary region, and point charge field) using the program Env by LePetit and Gelle.^55^ Two input files for Env (one for β-NaYF_4_:Ln^3+^ and one for LiYF_4_:Ln^3+^) are provided in the Supporting Information (section Env Input Files). All other input files are available at the RDR.

Information about the positions of the ions in the embedding scheme was written into an input file for the ORCA 6.0.1 program package, which was used to model the spectroscopic properties of the prepared clusters.^57–59^ To this end, state-averaged complete active space self-consistent field (SA-CASSCF) followed by N-electron valence state second-order perturbation theory (NEVPT2) and quasi-degenerate perturbation theory (QDPT) calculations for spin–orbit coupling (SOC) were performed for the prediction of wave functions and corresponding energies.^60^ All seven 4f-orbitals and the respective number of 4f-electrons for Er^3+^ (11), Tm^3+^ (12), and Yb^3+^ (13) were included in the active space. All states (multiplets) with all possible multiplicities were included in the state averaging. While SA-CASSCF provides a qualitative description of this multiplet structure and accounts for the static electron correlation, NEVPT2 captures a majority of the dynamical electron correlation that has not yet been accounted for through the mean-field treatment at the CASSCF level.^61–67^ Furthermore, the second-order scalar-relativistic Douglas–Kroll–Hess (DKH) Hamiltonian was employed to account for scalar-relativistic effects.^68–70^ Finally, SOC of the f-electrons was included via QDPT.^71^ Accurate treatment of SOC is absolutely crucial for the current investigation due to the semicore nature of the 4f-electrons and their high angular momentum.

Ln^3+^ and F^–^ in the quantum cluster were described by the all-electron SARC2-DKH-QZVP and DKH-DEF2-TZVPP basis sets, respectively.^72–74^ Calculations were accelerated by using the Resolution of Identity (RI) for the Coulomb integrals in conjunction with autogenerated fitting basis sets (AutoAux) and the chain-of-spheres approximation (COSX) for the exchange integrals with default grids.^75–77^ Capped point charges in the boundary region were represented by the charge of the respective ion plus the following ECP definitions: Li SD­(2,SDF),^78^ F SD­(2,MWB),^79^ Na SD­(10,SDF),^78^ and Y SD­(28,MWB).^80^

To simplify the setup of the active space, the newly developed atomic valence active space (AVAS) functionality of Orca was used. In case this failed, first, a CASSCF calculation for the Ln^3+^ with one iteration was performed. Afterward, the desired 4f-orbitals were rotated into the active space. Subsequently, a full CASSCF calculation was performed, delivering the required results. In a second step, the preoptimized Ln^3+^ was combined with the remaining parts of the cluster via the Orca mergefrag feature, followed by the full calculation as described above. The mergefrag functionality reliably ensured that the 4f-orbitals stay within the active space but required user involvement (different to AVAS). The resulting output files, also containing the Orca inputs, including the respective geometries, are available at the RDR. Input files for cluster 10 (relying on AVAS) and cluster 12 (making use of mergefrag) are available in the Supporting Information.

The ab initio emission spectra were generated from energy and intensity data by a custom program based on libcerf version 1.5–1.19.^81^ The stick spectra in the energy domain (cm^–1^) were convoluted with pure Gaussian functions with a full width at half-maximum of 20 cm^–1^. The simulated spectra were sampled with a resolution of 1 cm^–1^ in the energy domain, normalized to the largest signal, and subsequently transformed into the wavelength domain for comparison to the experimental photoluminescence spectra. The final simulated spectra were obtained as a weighted average over 14 individual spectra of the different clusters. The broadening parameter was estimated as the average from the two most intense lines of the experimental spectrum at 539.9 and 840.0 nm of β-NaYF_4_:Er^3+^. The raw data for the individual spectra are provided in the RDR.

## Methodology

### Chemicals

Yttrium oxide (Y_2_O_3_, 99.999%), erbium oxide (Er_2_O_3_, 99.99%), and thulium oxide (Tm_2_O_3_, 99.99%) were purchased from Alfa Aesar (Ward Hill, MA, U.S). Ytterbium oxide (Yb_2_O_3_, 99.998%), acetic acid (CH_3_COOH, > 99.7%), sodium oleate (CH_3_(CH_2_)_16_COONa, > 82%), ammonium fluoride (NH_4_F, > 98%), oleic acid (CH_3_(CH_2_)_7_CH=CH­(CH_2_)_7_COOH, 90%), and 1-octadecene (CH_2_=CH­(CH_2_)_15_CH_3_, 90%) were purchased from Sigma-Aldrich (Oakville, ON, Canada). Hexane (analytical grade), hydrochloric acid (HCl, 36.5%), and acetone (analytical grade) were purchased from Fischer Scientific (Waltham, MA, USA). Ethanol (99%) was purchased from Commercial Alcohols Inc. (Toronto, ON, Canada). All chemicals were used as received.

### Synthesis

The synthesis of UCNPs is briefly described in the following. Detailed information about all steps and quantities used is available in the Supporting Information (Synthesis of β-NaYF_4_:Ln^3+^UCNPs and Table S3). β-NaYF_4_ UCNPs (co)­doped with Er^3+^, Tm^3+^, and Yb^3+^ were synthesized following a synthesis route available in the literature.^82^ The particles were grown from the respective Y as well as Er, Tm, and Yb acetate precursors [Y­(Ac)_3_] and [Ln­(Ac)_3_]. These precursors were obtained by treating the required amounts of the analogue oxides with a one-to-one mixture of acetic acid and water overnight. The acetate precursors together with sodium oleate and ammonium fluoride as sodium and fluoride sources, respectively, and oleic acid and 1-octadecene as surfactants and solvents were then used to first create ultrasmall (sub-5 nm) sodium-deficient α-phase UCNPs. After isolating the resulting particles through repeated precipitation and washing, they were redispersed in oleic acid and octadecene and reheated to 300 °C. Large β-phase UCNPs grew at this temperature over the course of 3 h from the gradually dissolving α-phase particles. After the successful synthesis and isolation of the UCNPs from the reaction mixture by precipitation, the UCNPs were additionally washed with hexane and ethanol. The resulting UCNPs, ca. 800 mg per synthesis, were stored in hexane until further use. To obtain ligand-free UCNPs, the oleate ligands were removed from the surface of the nanoparticles by treatment with hydrochloric acid overnight (see Supporting Information for details).^83^

### Characterization

For the determination of the crystalline phase and phase purity via powder X-ray diffraction (XRD), a freshly stirred UCNP dispersion in hexane was dropped onto a glass slide and left to dry. Afterward, the nanoparticles were analyzed with a Bruker D8 Endeavor (Cu Kα, λ = 1.5401 Å) with the following settings: operating voltage and current of 44 kV and 40 mA with a step size of 0.02°, scanning speed of 1°·min^–1^, and scan range of 20–60° 2Θ. Transmission electron microscopy (TEM) was used to determine the morphology and size of the UCNPs, employing an FEI Tecnai Spirit microscope (operating at 120 kV). Therefore, a diluted sample of the UCNPs in hexane was drop-cast onto a Formvar and carbon coated film on top of a 300-mesh copper TEM grid. The resulting TEM images were analyzed with FIJI/ImageJ (U.S. National Institutes of Health, Bethesda, MA, USA). The obtained mean sizes and associated standard deviations were plotted with OriginPro 2021 (OriginLab Corporation, Northampton, MA, USA). Photoluminescence spectroscopy was performed on ligand-free UCNPs. Therefore, the nanoparticles were precipitated by adding an equal amount of acetone to the aqueous UCNP dispersion, followed by centrifuging the mixture for 5 min at a RCF of 8346*g*. After the supernatant was discarded, the resulting pellet was spread out onto a piece of aluminum foil (2 × 2 cm), covered by an appropriately sized glass slide, and then set to completely dry overnight. The as-prepared samples were mounted into the sample holder of a QuantaMaster 8075–21 spectrofluorometer from HORIBA Scientific (Burlington, ON, Canada) combined with a close-cycle helium cryostat CS202*E-DMX-1AL from Advanced Research Systems (Macungie, PA, USA), controlled by a LakeShore (Westerville, OH, USA) 335 temperature controller. Measurements were started once the samples were cooled to 20 K or lower. The spectrometer itself was equipped with double grating emission monochromators, a red-extended photomultiplier detector R13456 PMT (250 to 1010 nm), and a liquid nitrogen cooled InGaAs detector (800 to 1599 nm). The Er^3+^, Tm^3+^, and Yb^3+^-doped nanoparticles were excited using a 980 or 808 nm laser diode in continuous-wave mode with a maximum power density of 9.6 and 8.1 W·cm^–2^, respectively. The low-temperature emission spectra showing single multiplet transitions were obtained by averaging over three individual emission spectra with an integration time of 1 s per step each. Slit widths for the emission side were 0.5 nm or smaller, and step sizes were likewise 0.5 nm or smaller. All data obtained from the measurements were plotted using OriginPro.

## Results and Discussion

### Ordered LiYF_4_:Ln^3+^

#### Energy Levels

All clusters of LiYF_4_:Ln^3+^ were evaluated in terms of the energy levels of the central Ln^3+^ provided by the ab initio calculations. The resulting energy levels of Er^3+^ up to 20,700 cm^–1^ (≈485 nm, ^4^F_7/2_) are reported in Table 1. Energy levels for Tm^3+^ (up to 28,000 cm^–1^, ≈355 nm, ^1^D_2_) and Yb^3+^ are shown in Tables S1 and S2, respectively. Moreover, the full set of calculated energy levels, including all multiplets, for Er^3+^ and Tm^3+^ is provided in the RDR. The energy levels can be compiled in a separate file with the Python script *Calc_EnLev-OscStr.py*, also available there.

**Table 1:** Calculated (Ab Initio and Fitted) and Experimental Crystal Field Energy Levels of LiYF_4_:Er^3+^

crystal field energy level^a^ (^2S+1^L_J_)		this work (cm^–1^)	experiment^b^ ^34^ (cm^–1^)	fitted^35^ (cm^–1^)
^4^I_15/2_	1	0	0	–11
	3	41	17	8
	5	52	28	38
	7	79	57	60
	9	281	255	256
	11	324	290	298
	13	350	320	319
	15	365	355	352
^4^I_13/2_	1	6537	6540	6539
	3	6546	6545	6540
	5	6597	6585	6585
	7	6692	6680	6683
	9	6705	6704	6702
	11	6729	6731	6735
	13	6762	6745	6747
^4^I_11/2_	1	10264	10213	10215
	3	10282	10230	10230
	5	10355	10290	10284
	7	10359	10300	10305
	9	10375	10309	10312
	11	10402	10327	10323
^4^I_9/2_	1	12461	12364	12348
	3	12581	12486	12493
	5	12626	12540	12540
	7	12640	12568	12568
	9	12716	12663	12663
^4^F_9/2_	1	15414	15307	15306
	3	15441	15325	15325
	5	15452	15341	15343
	7	15527	15416	15418
	9	15555	15469	15471
^4^S_3/2_	1	18462	18433	18434
	3	18492	18492	18489
^2^H_11/2_	1	19562	19152	19170
	3	19574	19172	19192
	5	19617	19224	19233
	7	19684	19309	19303
	9	19697	19326	19313
	11	19719	19342	19329
^4^F_7/2_	1	20624	20571	20564
	3	20631	20573	20566
	5	20710	20662	20660
	7	20719	20671	20668
RMSE		155 (60)^c^		9

a The ^2S+1^L_J_ labels are derived from the state with the highest weight after the QDPT-SOC treatment. *M*_J_ values and irreducible representations for all levels are available in the work of Couto dos Santos et al.^34^

b The sample contained 1 mol % Er^3+^, but the obtained values should still be comparable as properties of the Ln^3+^ are fairly independent of the Ln^3+^ concentration in the low-percentage doping regime.^86^

c The value in brackets refers to the RMSE of only the quartet states (*S* = 4).

Compared to experimental values reported in the literature,^34,36–38^ all energy levels for the three different Ln^3+^ showed a reasonable match. The root-mean-square error (RMSE) for Er^3+^ was determined to be 155 cm^–1^, which was significantly larger than that for Tm^3+^ (64 cm^–1^) and Yb^3+^ (47 cm^–1^). The RMSE for Er^3+^ can be reduced to 60 cm^–1^ when the ^2^H_11/2_ multiplet is removed from the RMSE calculation. The energy of the ^2^H_11/2_ multiplet is overestimated by about 400 cm^–1^. This should possibly be corrected for simulations of Er­(-Yb) upconversion systems as the transitions from the ^2^H_11/2_, ^4^S_3/2_, and ^4^F_9/2_ multiplets to the ground state are the three most important ones.^11,12^ Nevertheless, the relative excitation energies of the NEVPT2 approach are of good quality. The varying quality of the multiplet energies, especially the ^2^H_11/2_ multiplet, is probably due to different capture of dynamic correlation, state interactions, relativistic effects, and failures of the perturbation theory in general. Also, a shift of 2% (overestimation of 400 cm^–1^ at around 19,250 cm^–1^) for the ^2^H_11/2_ multiplet is still quite good for this level of theory.^84,85^ Discrepancies for the other multiplets are less than 0.5%.

For Tm^3+^, a greater deviation between computational and experimental energy levels was observed with increasing distance to the ground state (Table S1). Interestingly, the RMSE of Tm^3+^ (64 cm^–1^) was remarkably close to the RMSE of Er^3+^ when the ^2^H_11/2_ multiplet was excluded (60 cm^–1^). Indeed, the RMSE for Yb^3+^ was also quite similar (47 cm^–1^, Table S2). A deviation of 60 cm^–1^ corresponds to approximately 6 nm at a wavelength of 1000 nm (10,000 cm^–1^) and 1.5 nm at a wavelength of 500 nm (20,000 cm^–1^), since the nanometer scale is inversely proportional to the wavenumber scale. Such an observation points toward the general accuracy of the setup, while also indicating a larger error of about 400 cm^–1^ for the ^2^H_11/2_ multiplet. In any case, the observed deviation should be kept in mind when employing calculated values where no reference data is available. For the ^2^H_11/2_ multiplet, similar errors were also observed when comparing computational and experimental data for β-NaYF_4_:Er^3+^ (see below). Overall, the RMSE of Yb^3+^ was the lowest among the three Ln^3+^ (47 cm^–1^, Table S2). However, since only two multiplets with rather small energetic separation are present and the number of energy levels used for calculating this error was rather low, the RMSE value might not be as significant as those for Er^3+^ and Tm^3+^.

Xiao et al. employed an adapted fitting procedure for the same dopant–host system to improve earlier predictions obtained with crystal field theory and generated energy levels with an RMSE of 9 cm^–1^ for Er^3+^ and Tm^3+^.^34,35,37^ Hence, their fit of energy levels was clearly superior to that of our ab initio results. However, obtaining the same precision is challenging when no fitting data are available. In this case, parameters must be taken from other structures or be obtained by a computational approach. An ab initio ansatz is possible for almost all systems where information about the crystal structure is available. It is also superior in the sense of adaptation. Once an input file has been designed, it can easily be adjusted for different structures (as demonstrated for β-NaYF_4_:Ln^3+^). Additionally, while we provided data for three different Ln^3+^ doped into LiYF_4_, the corresponding input files can easily be tuned to predict properties for the other Ln^3+^ ions. To the best of our knowledge, no other wave function-based approaches have been reported to date that predicted crystal field energy levels for LiYF_4_ doped with Er^3+^, Tm^3+^, or Yb^3+^.

Finally, for LiYF_4_, all Ln^3+^ are doped into sites with tetragonal *S*_4_ point group symmetry.^34^ For ions with an even number of electrons (i.e., Tm^3+^), this results in some degenerate energy levels beyond the regular Kramer’s degeneracy observed for ions with an odd number of electrons (i.e., Er^3+^ and Yb^3+^).^87^ This effect was correctly reproduced by our calculations, as seen, for example, for the ^3^F_4_ multiplet (Table S1). The states 2 and 3 as well as 8 and 9 of that multiplet shared the same energy. Therefore, the number of observable energy levels was reduced from seven to five as predicted by crystal field theory. The same effect was apparent in the reference data. That being said, the precise energetic order of the degenerate levels did not exactly follow our values, e.g., for the ^3^H_5_ multiplet.

#### Oscillator Strengths

In addition to the energy levels, the oscillator strengths between all crystal field energy levels were calculated. Table 2 lists (absorption) oscillator strengths obtained in this study in comparison to values from the literature for LiYF_4_ doped with (A) Er^3+^, (B) Tm^3+^, and (C) Yb^3+^.^39,40^ The multiplet oscillator strengths calculated in this work were obtained by first averaging the oscillator strengths to a given excited state over the ground state multiplet components (crystal field energy levels). All components were assumed to be equally populated. These averaged oscillator strengths were then added across the excited state multiplet components. All oscillator strengths involving higher multiplets can be generated with the Python script *Calc_EnLev-OscStr.py* and the output file of the respective Ln^3+^ (both available in the RDR). Results for the individual crystal field oscillator strengths are also available there.

**Table 2:** Calculated (Ab Initio and Fitted) and Experimental Absorption Multiplet Oscillator Strengths between the Ground State and Higher Multiplets of (A) Er^3+^, (B) Tm^3+^, and (C) Yb^3+^ Doped into LiYF_4_ ^a^

(A) Er^3+^					
^4^I_15/2_ →	this work	ref 39 ^b^		ref 40 ^c^	
		experiment	fitted	experiment	fitted
^4^I_13/2_	0.96	1.54	0.95	0.72	0.70
^4^I_11/2_	0.28	0.42	0.41	0.16	0.31
^4^I_9/2_	0.01	0.15	0.17	0.06	0.04
^4^F_9/2_	0.51	1.23	1.35	0.61	0.62
^4^S_3/2_	0.28	0.34	0.37		
^2^H_11/2_	0.14	2.36	2.68	1.29	1.26
^4^F_7/2_	0.88	1.01	1.42	0.47	0.93

(B) Tm^3+^	
^3^H_6_ →	this work
^3^F_4_	0.11
^3^H_5_	0.64
^3^H_4_	0.52
^3^F_3_	0.82
^3^F_2_	0.26
^1^G_4_	0.02
^1^D_2_	0.20

(C) Yb^3+^	
^2^F_7/2_ →	this work
^2^F_5/2_	0.98

a The oscillator strengths were multiplied by 1 × 10^6^.

b These values were obtained by averaging over four samples with an Er^3+^ concentration of 4.9 mol %, 13.6 mol %, 21.4 mol %, and 41.3 mol %, and no concentration effects were observed.

c The sample contained 1 mol % Er^3+^.

Two data sets for LiYF_4_:Er^3+^ were available in the literature (Table 2A) for benchmarking the values calculated by the ab initio model.^39,40^ Interestingly, these two data sets differed by up to a factor of 3 for individual multiplet oscillator strengths. However, they followed the same trend in terms of the relative strength among the multiplets. Our calculated values mostly fell between the two sets of values reported in the literature. Their relative strengths also followed the trend observed previously. An exception was the ^2^H_11/2_ multiplet, the oscillator strength of which was off by an order of magnitude. Thus, in general, the wave function for the ^2^H_11/2_ multiplet appeared to converge poorly. Consequently, all derived properties suffered from the same shortcoming. The values fitted from the experimental data seem therefore a better choice for the oscillator strength of the ground to ^2^H_11/2_ multiplet excitation.

Experimental oscillator strengths for LiYF_4_ doped with either Tm^3+^ or Yb^3+^ are not available in the literature. However, oscillator strengths for LiYF_4_:Er^3+^ provided reliable data for multiplets with correctly predicted energy levels (Tables 1 and 2). Moreover, energy levels for Tm^3+^ and Yb^3+^ (Tables S1 and S2, respectively) were in good agreement with the measured values. Therefore, the values listed in Table 2B,C are expected to accurately predict the oscillator strengths or, at least, the trends between the different multiplets.

Finally, oscillator strengths were also calculated for LiYF_4_ doped with Er^3+^ as well as Yb^3+^ using the setup with the larger quantum cluster (see above). Values differed by at most 5% and on average by 3% compared to the setups with the smaller quantum cluster. Hence, a larger quantum cluster is not expected to drastically correct the energy levels or oscillator strengths. The respective data are provided in the RDR.

Overall, the general trend for the ab initio results of LiYF_4_:Ln^3+^ was found to be quite positive. Crystal field energy levels and oscillator strengths were reproduced with good accuracy. This paves the way for examining the equivalent properties for a disordered crystal.

### Disordered β-NaYF_4_:Ln^3+^

#### Averaging Scheme

Given its ordered structure with only one possible configuration per supercell, the analysis of data obtained for LiYF_4_:Ln^3+^ was straightforward. Conversely, the disordered character of β-NaYF_4_:Ln^3+^ required the development of a method that involves averaging over the results obtained for the different clusters. This was addressed by introducing a weighting factor to each cluster that reflects the probability of finding the specific ion arrangement of that cluster in the material (Table 3). This weighting factor is comprised of two parts: the statistical occurrence (degeneracy of the configuration) and the occupation probability (from the relative energy) of the cluster. A general overview that describes the steps required for obtaining the weighting factors is provided hereafter. The exact numbers for the calculation and the resulting factors can be found in the file *Energy-Levels_NaYF4-b_Ln* in the RDR.

**Table 3:** Calculation of Weighting Factors for the β-NaYF_4_:Er^3+^ Clusters^a^

cluster *n*, (Na^+^:Y^3+^ distribution)	degeneracy (deg.)	statistical occurrence (deg./64·*x*/3)	Δ*E* = *E*_*n*_ – *E*_10_ (kJ·mol^–1^)	Boltzmann distribution^b^ *f*(*E*_*n*_)/*f*(*E*_10_)	weighting factor (%)^c^ (statistical occurrence Boltzmann distribution)
Wyckoff site: 1f		(*x* = 1)			
1 (2:0)	1	0.08	0.10	0.98	12.9
2 (1:1)	2	0.17	2.36	0.61	16.0
3 (0:2)	1	0.08	3.38	0.49	6.5
Wyckoff site: 1a		(*x* = 2)			
5 (5:1)	6	0.06	4.57	0.38	3.8
6 (4:2)	3	0.03	3.37	0.49	2.4
7 (4:2)	6	0.06	1.82	0.68	6.7
8 (4:2)	6	0.06	1.79	0.69	6.8
9 (3:3)	2	0.02	1.23	0.77	2.5
10 (3:3)	6	0.06	0.00	1.00	9.8
11 (3:3)	12	0.13	1.60	0.71	14.1
12 (2:4)	6	0.06	1.92	0.67	6.6
13 (2:4)	6	0.06	2.29	0.62	6.1
14 (2:4)	3	0.03	4.79	0.37	1.8
15 (1:5)	6	0.06	4.21	0.41	4.1

a The table groups the clusters according to the site at which they are centered, i.e., the 1f site (clusters 1 to 3) and the 1a site (clusters 5 to 15). *E* in the table refers to the energy of the underlying supercell as calculated in our previous work.^29^ The energies (*E*) were obtained from supercells optimized by means of DFT with periodic boundary conditions.^29^

b *T*_syn_ = 300 °C, the temperature at which the nanoparticles for this study were prepared.

c Similar values were obtained for Tm^3+^ and Yb^3+^. Detailed information for all three Ln^3+^ is available in the file *Energy-Levels_NaYF4-b_Ln* in the RDR.

The statistical occurrence reflects the fact that not all clusters are equally likely from stochastic considerations. When calculating its value, it was assumed that all disordered sites are independent of each other, and only the total composition of the respective cluster must stay constant. For example, there are six different possibilities of arranging five Na^+^ and one Y^3+^ at the six disordered 1f sites around an Ln^3+^ doped into a 1a site (first and second column of Table 3). This arrangement was denoted as a 5:1 distribution. A 1:5 distribution involves one Na^+^ and five Y^3+^. Indeed, it does not matter at which exact position the single ion is placed. Hence, all six arrangements were combined into one 6-fold degenerate cluster (cluster 5 for a 5:1 distribution and cluster 15 for the 1:5 distribution). Having said this, for a 4:2 (and 2:4) distribution, already 15 different arrangements exist. Also, for these and the other remaining distributions, two additional aspects must be considered. First, the location of the two Y^3+^ matters for the surroundings of the central ion, being either located in the same column along the *c*-axis or in two separate ones. Second, if these Y^3+^ ions are not in the same column, they can share the same c-coordinate or not. To account for these two aspects, the 15 arrangements were subdivided into three different clusters. These clusters were labeled 6, 7, and 8 for the 4:2 distribution, as well as 12, 13, and 14 for the 2:4 distribution, respectively (Table 3). Further, a degeneracy was assigned to each cluster. This scheme was once more repeated for a 3:3 distribution, resulting in three different clusters (9, 10, and 11) with their respective degeneracies. Finally, for the cluster centered at a 1f site, only two 1f disordered sites are part of the first anionic coordination sphere (Figure 3A). The distribution of Na^+^ and Y^3+^ at these sites was dissected into three cases, i.e., a 2:0, 0:2, and 1:1 distribution (clusters 1, 2, and 3, respectively), with the latter being doubly degenerate.

Once the number of degenerate configurations for each cluster was established, it was divided by the overall number of configurations for this site, i.e., by 4 for a 1f-doped site and 64 for the 1a-doped site. Subsequently, this number was multiplied by a factor of 1/3 and 2/3 for the 1f and 1a site, respectively, representing the relative doping probability of an Ln^3+^ at this site. This is because in the β-NaYF_4_ crystal, there are half as many 1f sites occupied by Y^3+^ as there are 1a sites occupied by Y^3+^ (Figure 1A). A Ln^3+^ can only be doped into sites occupied by Y^3+^. Ultimately, this resulted in the statistical occurrence for each cluster, being the first factor to be used in the averaging scheme (Table 3, third column). It must be pointed out that the disorder at the 2h sites was not considered for this scheme, even though energetic differences for supercells with different 2h disorder are less pronounced than for the ones with 1f disorder.^29^ It has previously been shown that for a 2 × 2 × 4 supercell, several hundreds of millions of configurations are possible when accounting for both disordered sites at the same time.^29^ Instead, additional calculations were conducted to specifically investigate the influence of the disorder at the 2h sites (see below).

The second factor included in the averaging scheme was the occupation probability for each cluster. This factor is derived from the Boltzmann distribution since the supercells, from which the respective clusters were created, exhibited varying total energies. These energetic differences (Table 3, fourth column) led to different probabilities of forming the underlying supercell and, thus, the respective cluster. The Boltzmann distribution was calculated as

1 $$\frac{f\left(E_{n}\right)}{f\left(E_{10}\right)}=\exp\left(-\Delta E_{n}\cdot {\left(k_{B}\cdot N_{A}\cdot T_{syn}\right)}^{-1}\right)$$

with *f*(*E*_*n*_)/*f*(*E*_10_) being the occupation probability of the *n*th cluster relative to that of cluster 10, *k*_B_ the Boltzmann constant, *N*_A_ the Avogadro constant, and *T*_syn_ the synthesis temperature. Here, *T*_syn_ refers to the temperature at which the particles were synthesized. *T*_syn_ determines the energy available in the system and, therefore, the ability of the system to overcome energetic differences between the available clusters. Temperatures used in later calculations instead refer to the condition under which the particles were optically characterized. Additionally, during the synthesis of UCNPs, entropic and kinetic effects play critical roles, which were not accounted for here. However, the energetic difference by itself will be an influential parameter, which was accounted for. The calculated occupation probabilities obtained from these energetic differences make up the second part of the weighting scheme (Table 3, fifth column).

Ultimately, for each cluster, the statistical occurrence and occupation probability were multiplied and then expressed as a percentage relative to all of the weighting factors. The last column of Table 3 represents this value. Preliminary results for clusters 4 and 16 (representing a 6:0 and 0:6 distribution, respectively) indicated very low weighting factors. Therefore, these clusters were omitted from the calculations.

Given the few different clusters of the 1f site, cluster 2 (Figure 3A) had the highest weighting factor. Interestingly, the second in line is cluster 11. This is surprising as cluster 10 represents the go-to configuration when treating β-NaYF_4_(:Ln^3+^) as an ordered crystal consisting of a single configuration and as the configuration with the lowest energy. If this single cluster was an appropriate representative of the structure overall, its weighting factor is expected to be much higher. These results indicate that designing a crystal structure of β-NaYF_4_ for theoretical calculations solely based on the supercell of cluster 10 might not yield results that are representative of the whole crystal. Rather, averaging over multiple clusters using weighting factors, as proposed in this work, is required to provide a more complete picture.

#### Energy Levels

Once the weighting factors were established, the weighted average of all clusters with respect to the weighting factors was calculated for the crystal field energy levels and oscillator strengths (see below). The resulting energy levels for β-NaYF_4_ doped with (A) Er^3+^, (B) Tm^3+^, and (C) Yb^3+^ are presented in Table 4. Energy levels up to 20,800 cm^–1^ (≈485 nm, ^4^F_7/2_) and 28,000 cm^–1^ (≈355 nm, ^1^D_2_) are displayed for Er^3+^ and Tm^3+^, respectively. The energy levels for the individual clusters are provided in the RDR (*Energy-Levels_NaYF4-b_Ln*). The full set of calculated energy levels (for all multiplets and all clusters) is available in the respective output files in the RDR and can be extracted using the Python script also deposited there.

**Table 4:** Calculated (Ab Initio) Crystal Field Energy Levels and Experimental Multiplets as well as Fitted Crystal Field Energy Levels (as Available in the Literature) of β-NaYF_4_ Doped with (A) Er^3+^, (B) Tm^3+^, and (C) Yb^3+^

(A) Er^3+^				
crystal field energy level (^2S+1^L_J_)		this work (cm^–1^)	experiment^32^ ^a^ (cm^–1^)	fitted^33^ ^b^ (cm^–1^)
^4^I_15/2_	1	0	0	0
	3	81		2
	5	136		4
	7	194		61
	9	242		72
	11	294		79
	13	340		79
	15	411		152
^4^I_13/2_	1	6586	6636	6519
	3	6619		6520
	5	6644		6521
	7	6676		6550
	9	6702		6553
	11	6737		6569
	13	6799		6625
^4^I_11/2_	1	10312	10226	
	3	10334		
	5	10353		
	7	10370		
	9	10400		
	11	10439		
^4^I_9/2_	1	12542	12419	
	3	12562		
	5	12639		
	7	12683		
	9	12739		
^4^F_9/2_	1	15450	15310	15170
	3	15477		15191
	5	15505		15220
	7	15531		15246
	9	15566		15265
^4^S_3/2_	1	18507	18529	18440
	3	18536		18493
^2^H_11/2_	1	19609	19229	19308
	3	19644		19314
	5	19661		19321
	7	19685		19326
	9	19704		19334
	11	19723		19343
^4^F_7/2_	1	20622	20524	
	3	20675		
	5	20731		
	7	20768		

(B) Tm^3+^					
crystal field energy level (^2S+1^L_J_)		this work (cm^–1^)	crystal field energy level (^2S+1^L_J_)		this work (cm^–1^)
^3^H_6_	1	0	^3^H_4_	1	12546
	2	9		2	12566
	3	54		3	12588
	4	80		4	12613
	5	122		5	12642
	6	149		6	12673
	7	187		7	12721
	8	223		8	12766
	9	246		9	12776
	10	296			
	11	314	^3^F_3_	1	14279
	12	405		2	14317
	13	417		3	14341
				4	14361
^3^F_4_	1	5634		5	14380
	2	5661		6	14398
	3	5689		7	14420
	4	5717			
	5	5730	^3^F_2_	1	14936
	6	5750		2	14951
	7	5761		3	15018
	8	5782		4	15061
	9	5823		5	15084
^3^H_5_	1	8207	^1^G_4_	1	21188
	2	8218		2	21222
	3	8267		3	21250
	4	8288		4	21300
	5	8317		5	21323
	6	8348		6	21351
	7	8377		7	21384
	8	8428		8	21446
	9	8440		9	21502
	10	8528			
	11	8539	^1^D_2_	1	27961
				2	27971
				3	28056
				4	28115
				5	28137

(C) Yb^3+^					
crystal field energy level (^2S+1^L_J_)		this work (cm^–1^)	crystal field energy level (^2S+1^L_J_)		this work (cm^–1^)
^2^F_7/2_	1	0	^2^F_5/2_	1	10126
	3	120		3	10265
	5	244		5	10466
	7	416			

a The sample contained 10 mol % Er^3+^ and 20 mol % Y^3+^ and values represent the position of the multiplet at maximum intensity.

b The authors fitted the values from magnetization data, not optical spectra. Only a few multiplets were predicted.

Comparison of our data obtained for the crystal field energy levels of Er^3+^ with the positions of the maximum emission intensity of the corresponding multiplet as reported by Zhang et al. unveiled a satisfactory match (Table 4A),^32^ though the different chemical composition of the sample by Zhang et al., i.e., β-NaYF_4_ codoped with 10 mol % Er^3+^ and 20 mol % Yb^3+^, should be noted. Moreover, the maximum intensity was not necessarily found at the barycenter of the multiplet. Therefore, this comparison should be treated as a first indication of the reliability of the ab initio results and not as proof of their qualitative accuracy. Similar to LiYF_4_:Er^3+^, prediction of the position of the ^2^H_11/2_ multiplet resulted again in significantly higher energies than those obtained experimentally. Unfortunately, to the best of our knowledge, no other data on experimental crystal field levels for any of the three lanthanide ions are available in the literature. Also, Er^3+^ is the only lanthanide for which theoretical data for at least some crystal field energy levels was reported.^33^ That being said, good agreement with this data set was observed (Table 4A).

To allow for better comparison of ab initio and experimental data, several UCNPs (co)­doped with Er^3+^, Tm^3+^, and Yb^3+^ in various concentrations were synthesized following a procedure reported by Rinkel et al. (see Experimental Details and Supporting Information).^82^ The single-doped samples (i.e., samples ^4.2^Er and ^4.2^Tm) almost exactly matched the simulated particles in the embedding model in terms of dopant concentration. The codoped samples (i.e., samples ^2.1^Er/^2.1^Yb, ^2.1^Er/^2.1^Tm, ^0.5^Tm/^3.7^Yb, and ^0.5^Tm/^20^Yb) were synthesized as they allowed for more flexible excitation wavelength choice and thus the observation of additional transitions, otherwise not possible due to overlap between excitation and emission wavelength, for example, the ^4^I_11/2_–^4^I_15/2_ transition of Er^3+^ at 980 nm, the ^2^F_5/2_–^2^F_7/2_ transition of Yb^3+^ at 980 nm, and the ^3^H_4_–^4^H_6_ transition of Tm^3+^ at 820 nm.

An overview of all synthesized samples, including their chemical compositions and sizes, is given in Table S3. X-ray powder diffraction (XRD) analysis confirmed that all UCNPs crystallized in the hexagonal (β) phase of NaYF_4_ (Figure S1). Transmission electron microscopy (TEM) images showed monodisperse nanoparticles with sizes ranging from 57 to 109 nm and narrow size distribution (Figures S2 and S3). The nanoparticles exhibited a characteristic morphology of hexagonal platelets. Photographs taken with a smartphone camera of five of the six samples under excitation with a hand-held 980 nm laser are shown in Figure 4A, showing the characteristic green, red, and blue upconversion emission of Er^3+^ and Tm^3+^-doped UCNPs. The purely Tm^3+^-doped UCNPs did not show any visible emission, neither under 980 nor 808 nm excitations with the hand-held laser diodes. This was to be expected, given the lack of Yb^3+^ as a codopant acting as a sensitizer. Moreover, it has been shown that for high Tm^3+^ dopant concentration (such as our 4.2 mol %), cross-relaxation processes often result in the loss of emission intensity.^88,89^

**Figure 4:**
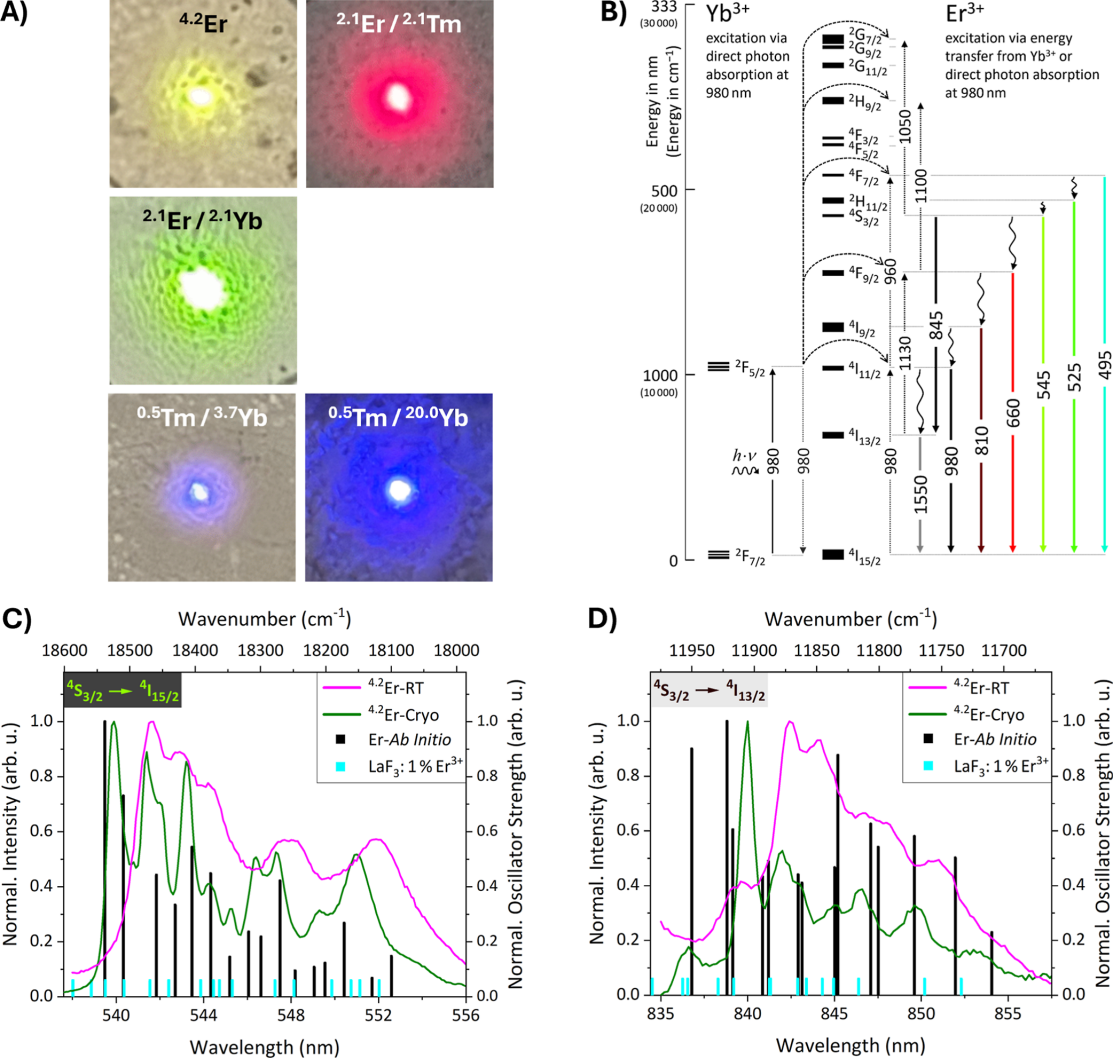
(A) Photographs taken with a smartphone camera of various β-NaYF_4_:Ln^3+^ UCNPs deposited on a glass slide (ca. 2 × 2 cm) upon excitation with a hand-held 980 nm laser. (B) Energy level diagram showing probable upconversion and downshifting pathways for Er^3+^ together with Yb^3+^ under 980 nm excitation. (C,D) Photoluminescence spectra recorded at room temperature (color code magenta) as well as 20 K (color code olive) of β-NaYF_4_ UCNPs doped with 4.2 mol % Er^3+^ showing the (C) ^4^S_3/2_ → ^4^I_15/2_ and (D) ^4^S_3/2_ → ^4^I_13/2_ transition, respectively. Excitation wavelength (λ_ex_) = 980 nm; power density = 9.6 W·cm^–2^. Vertical bars in black represent the averaged positions of the crystal field transitions of the respective multiplets according to ab initio calculations. The heights of the bars are equal to the averaged oscillator strengths for the respective crystal field transitions. Vertical bars in cyan represent the positions of crystal field transitions obtained from experimental literature data for LaF_3_:1 mol % Er^3+^.^30^ The heights of these bars were chosen arbitrarily.

Low-temperature and room temperature photoluminescence spectra of the synthesized UCNPs, exhibiting the characteristic upconversion and downshifting emission bands of Er^3+^, Tm^3+^, and Yb^3+^ under 980 or 808 nm excitation, are provided in Figures S4–S6. An overview of the samples and color codes for the spectra recorded at the different temperatures is provided in Table S4. The origins of the mentioned transitions are shown in detail in the energy level and energy transfer diagram for Er^3+^–Yb^3+^ codoped systems in Figure 4B. Partial energy level diagrams for Tm^3+^ and Yb^3+^ are shown in Figures S7 and S8.

The following discussion of experimental spectra in comparison with theoretical data will focus on the photoluminescence spectra of β-NaYF_4_ UCNPs (co)­doped with Er^3+^. The reason for this is that Er^3+^, with its uneven number of electrons and due to a lack of a magnetic field, features multiplets that split into a small number of crystal field energy levels. This is especially true for the ^4^S_3/2_ multiplet (two Stark sublevels), which at the same time is also one of the main emitting states of Er^3+^-doped UCNPs. This reduced the number of observable crystal field transitions. Additionally, the relatively high emission intensity made it possible to resolve individual transitions in the recorded signal. On the other hand, most multiplets of Tm^3+^ split into many more crystal field energy levels, and thus, many more transitions form the observed signal. Resolving the multiplet into individual crystal field transitions was therefore not achievable.

Intensity-normalized low-temperature spectra of Er^3+^-doped samples ascribed to the respective Er^3+^ f–f transitions are depicted in Figure S9. The analogous spectra for samples (co)­doped with Tm^3+^ and Yb^3+^ are provided in Figures S10 and S11, respectively. The exact upconversion and population dynamics and shapes of the spectra are not relevant for the comparison with the theoretical data as discussed in the following. Indeed, as expected, changes with respect to spectral shape and position between the differently doped samples were minor. The observed similarities conveniently allow for a transfer of knowledge gained from these samples to other UCNPs with similar doping concentrations—particularly to those that are most commonly considered in the field, i.e., 2 mol % for Er^3+^, 20 mol % for Yb^3+^, and 0.5 mol % for Tm^3+^.^11,12^

None of the recorded photoluminescence spectra provided clearly separated crystal field transitions. This is because all crystal field transitions of the involved multiplets for a specific transition overlap (especially for multiplets with large *J* values). Additionally, the disordered lattice results in a variety of transitions from individual Ln^3+^. Both effects combined result in the observed spectra. Indeed, to still prove that the theoretical results are a good fit, the computed transition positions were added as black bars to each spectrum in Figures S9 and S10 as well as Figure S11 for Er^3+^ and Tm^3+^ as well as Yb^3+^, respectively. It is important to note that these transition positions represent the weighted average over all clusters instead of individual clusters. For most transitions, the experimental emission range and theoretical positions showed good agreement for all three Ln^3+^. For the ^2^H_11/2_–^4^I_15/2_ transition of Er^3+^, Figure S5A, however, the predicted transition positions shifted toward higher wavenumbers by about 350 cm^–1^. This is in line with the observation that the ^2^H_11/2_ multiplet is predicted at energies that are too high. The difference between the emission range of the experimental photoluminescence spectra and the calculated transition wavenumbers for all other multiplets and those of Tm^3+^ and Yb^3+^ was in the order of 60 cm^–1^ (1.5 nm at 500 nm, 6 nm at 1000 nm). The discrepancy between the data sets is, therefore, similar to the discrepancy reported above for LiYF_4_:Ln^3+^.

While it was not possible to resolve all individual crystal field transitions for any transition, for two transitions of Er^3+^, the number of crystal field energy levels involved was low enough to almost identify the expected number of crystal field transitions (peaks). Especially in the case of spectra recorded from β-NaYF_4_ UCNPs doped with 4.2 mol % Er^3+^ at low temperature (olive line in Figure 4C,D), the expected 16 peaks of the ^4^S_3/2_ → ^4^I_15/2_ transition and 14 peaks of the ^4^S_3/2_ → ^4^I_13/2_ transition could mostly be identified upon close inspection. Added to Figure 4C,D are the average positions of the respective crystal field transitions as black bars, together with their averaged oscillator strengths as the height of the bars according to the ab initio calculations. Their positions and heights mostly overlap with the positions of the experimentally observed peaks. This constitutes a first indication of the validity of the proposed disordered model.

Room temperature photoluminescence spectra for the ^4^S_3/2_ → ^4^I_15/2_ and ^4^S_3/2_ → ^4^I_13/2_ transition are depicted in Figure 4C,D, respectively. Both room temperature spectra are shifted by several nanometers to longer wavelengths compared to the respective low-temperature spectra. The significant peak shift of Er^3+^ upon cooling has been reported previously and was ascribed to the contraction of the crystal lattice at low temperature.^90,91^ This explanation fits nicely to the observation that the low-temperature peaks were of better match with the ab initio results, since the DFT crystal structure optimizations, on which these ab initio calculations were based on, did not incorporate temperature-induced effects.^29^

Overall, the computationally predicted and averaged crystal field transitions were in very good agreement with the experimental results. Furthermore, the transitions calculated at an ab initio level of theory also matched the low-temperature spectra in Figure 4C,D better than do the experimental transitions measured for Er^3+^ doped into a different host material (LaF_3_).^30^ This underlines the predictive power of the computational model presented in this work. Moreover, the averaged relative oscillator strengths matched the experimental values well, which are discussed further below.

#### Influence of the 1f-Disorder

So far, only averaged transition positions and averaged oscillator strengths from the ab initio calculations were compared to the respective spectra. Averaging of the results for the different clusters produces clean data sets that can easily be displayed and compared to other data sets. However, the “mean structure” that is invoked by the averaging does in fact not exist as the individual clusters do not interconvert on the time scale of the experiment. For truly meaningful comparisons with the experimental data, the contribution of each cluster needs to be accounted for individually, while retaining the weighting factors for the respective clusters. Such an approach can be insightful, for example, when predicting an emission spectrum for a disordered structure.

To this end, the low-temperature photoluminescence spectra shown in Figure 4C,D were combined with the predicted results for the transition positions from each cluster individually (Figure S12A,B, respectively). Herein, the short semitransparent bars represent the individual transition positions. A color code was applied so that each bar can be retraced to its respective crystal field transition (Figure S13). To also give a sense of the oscillator strengths of the individual transitions, the large bars from Figure 4 were retained (recolored large opaque bars in Figure S12).

The height of a large bar therefore represents the oscillator strength of all small bars of the same color. The heights of the small bars instead represent their weighting factors.

In Figure S12A,B, the influence of the 1f-disordered sites on the positions of the crystal field transitions is clearly visible. The transitions are spread considerably along the whole emission range of the experimental spectra for the respective multiplet transitions. Indeed, they reproduce the experimental emission range rather accurately. The experimental spectra should therefore also be the result of an overlap of all the individual contributions. This could also explain the inability to resolve the peaks of the experimental spectra further. To test this hypothesis, individual crystal field oscillator strengths were assigned to the individual crystal field transitions and, together with the transition positions, used for simulating the respective emission spectra.

#### Crystal Field Oscillator Strengths

With the information about the individual clusters available, both their energy levels, and the oscillator strengths between them, it was possible to reproduce the experimental spectra to a very satisfying degree. For each cluster, a spectrum was created by convoluting the crystal field transitions and oscillator strengths with pure Gaussian functions. Next, a single spectrum was received by averaging the individual spectra. Finally, the ab initio spectra were shifted and the theoretical spectra shown in Figure 5A,B (black line) were obtained. When generating the ab initio spectra, it was assumed that the two crystal field levels of the ^4^S_3/2_ multiplet had equal population (= 1). Note that it is not straightforward to select a specific temperature for a sample that, on one hand, is cooled to 20 K using a cryostat setup and, on the other hand, exhibits excitation laser-induced heating (excitation power density: 10 W·cm^–2^). To account for such temperature effects, future investigations need to probe whether a different temperature and, therefore, Boltzmann population might be more appropriate. The spectral shift was introduced so that the relative separation of the crystal field transitions and the overlap between the experimental and ab initio spectra could be observed better. The ab initio spectra in Figure 5A,B were shifted based on the first dominant transition of each set of spectra. Details on the values for the different clusters can be found in the files *Spectrum_NaYF4-b_Er-545nm* and *Spectrum_NaYF4-b_Er-845nm* in the RDR.

**Figure 5:**
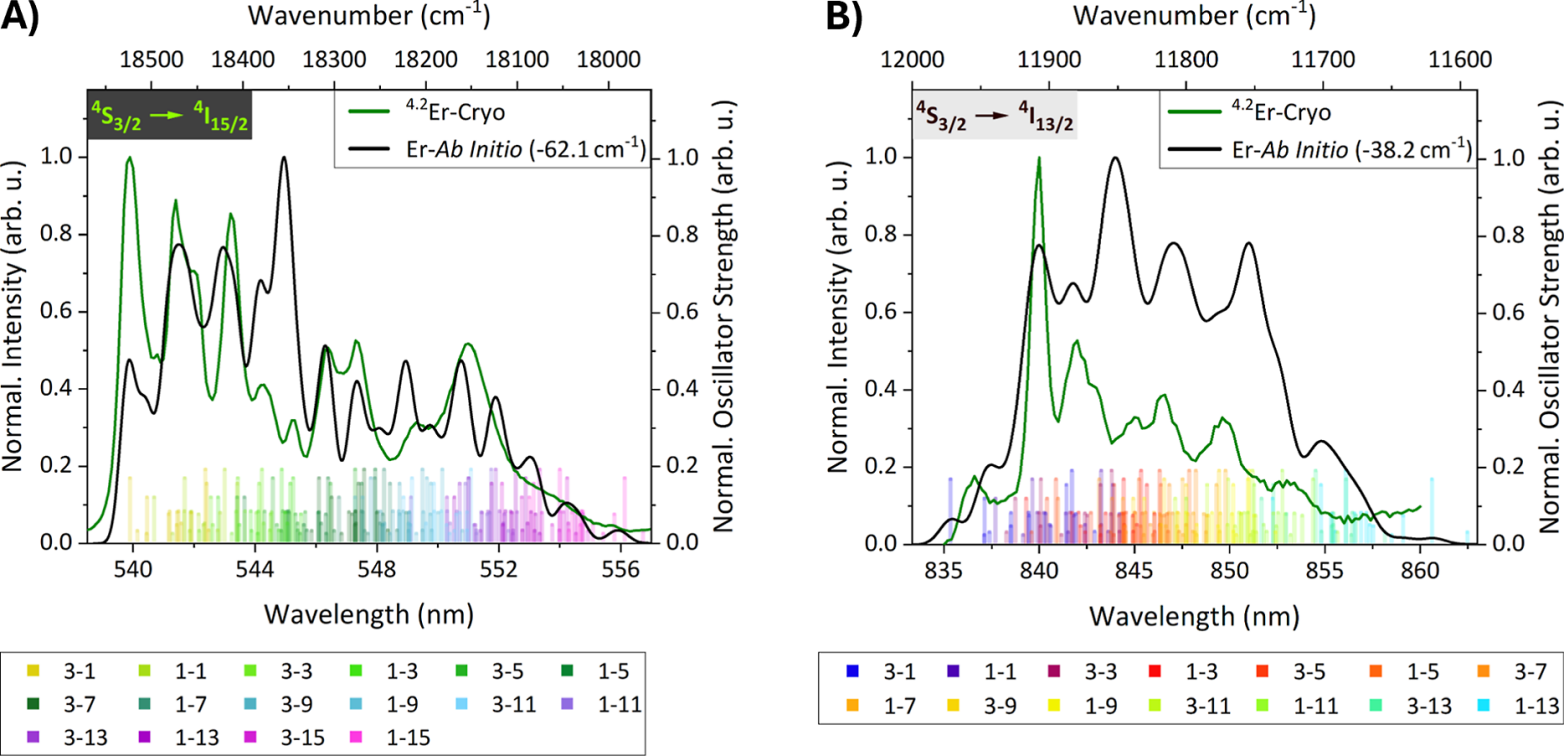
Low-temperature photoluminescence (20 K, color code olive) and ab initio spectra (color code black) of Er^3+^-doped β-NaYF_4_ for the (A) 545 nm (^4^S_3/2_ → ^4^I_15/2_) and (B) 845 nm (^4^S_3/2_ → ^4^I_13/2_) transition. The ab initio spectra in (A,B) were shifted by −62 cm^–1^ and −38 cm^–1^, respectively. Small semitransparent bars indicate the positions of the crystal field transitions for individual clusters. Each of the 14 clusters generates (A) 16 and (B) 14 individual transitions. Their labeling in the legend follows the scheme *n*_1_-*n*_2_" where *n*_1_ indicates the crystal field energy level of the initial multiplet (^4^S_3/2_) and *n_2_* that of the final multiplet (^4^I_15/2_). The height of the smaller bars represents the weighting factor of the cluster they originate from (not their oscillator strength), and bars with the same height were retrieved from the same cluster.

The theoretical spectra showed promising agreement with the experimental ones after slightly shifting them to lower wavenumbers. These shifts correspond to less than 0.4% of the band's average wavelengths and are motivated by the discrepancy between experimental and calculated results discussed above. Shifting the spectrum allows observation of the remarkable overlap between the computed and measured spectra on a relative scale. All transition energies were shifted by a constant value; therefore, the shape of the calculated spectrum stayed the same. As mentioned above, a shift of less than 1% for the assessed system is still quite good for this level of theory.^84,85^

The crystal field oscillator strengths are similarly predicted qualitatively correct, although slight misalignments still exist for some of the peaks. It is important to note that the crystal field oscillator strengths (but also the crystal field energy levels) seem to be highly susceptible to the ion arrangement in the disordered lattice. Their specific values for the oscillator strengths varied significantly from cluster to cluster, by more than 1 order of magnitude. Detailed information is available in the file *Oscillator-Strengths_NaYF4-b_Ln* in the RDR. To visualize this sensitivity to the environment, the low-temperature emission spectrum of the ^4^S_3/2_ → ^4^I_15/2_ transition for β-NaYF_4_:Er^3+^ and its ab initio counterpart of the averaged results are combined with ab initio spectra for individual clusters (Figure S14A). In particular, the spectra of clusters 2 and 11, with the two highest weighting factors, and cluster 10, commonly used in other works, are displayed in this figure. The individual spectra predicted transitions at energies that are too high or low, left gaps where none are in the experimental emission spectrum, and misrepresented the respective oscillator strengths. Thus, results from the individual clusters did not provide theoretical spectra with similar agreement to the experimental data, and only the averaged result provided a good fit.

Overall, the crystal field energy levels and oscillator strength from the ab initio results showed a crucial dependency on the actual positions of the F^–^ around the Ln^3+^. Including disorder into a computational model was therefore identified as a prerequisite for obtaining realistic predictions of the photoluminescence spectra. These findings lend great credibility to the computational model but also the setup including disorder.

#### Multiplet Oscillator Strengths

Because of the way the experimental photoluminescence spectra were recorded, relative intensities of different multiplets cannot be directly compared to the computed oscillator strengths. The population of the initial state (the excited multiplet) in an upconversion emission spectrum is unknown and is unlikely to be equal among the different excited multiplets involved. Indeed, oscillator strengths are therefore often determined from absorption spectra, where transitions originate from the ground state (multiplet).^32^ Therefore, the calculated multiplet (absorption) oscillator strengths for Er^3+^ were compared to those of a data set available in the literature. Table 5 lists multiplet absorption oscillator strengths of β-NaYF_4_ doped with (A) Er^3+^, (B) Tm^3+^, and (C) Yb^3+^ calculated in this work and taken from the literature for comparison.^32^ The multiplet oscillator strengths were calculated as described for LiYF_4_. Additional values for the transitions not involving the ground state multiplet can be found in the file *Oscillator-Strengths_NaYF4-b_Ln* in the RDR. All remaining oscillator strengths (involving higher multiplets) can be generated with the Python script *Calc_EnLev-OscStr.py* and the output file of the respective Ln^3+^ (both available in the RDR). Oscillator strengths of all crystal field transitions for all clusters are also available there.

**Table 5:** Calculated (Ab Initio and Fitted) and Experimental Absorption Oscillator Strengths between the Ground State and Higher Multiplets of β-NaYF_4_ Doped with (A) Er^3+^, (B) Tm^3+^, and (C) Yb^3+^ ^a^

(A) Er^3+^			
^4^I_15/2_ →	this work	experiment^32^ ^b^	fitted^32^ ^b^
^4^I_13/2_	1.01	1.33	0.86
^4^I_11/2_	0.30		0.40
^4^I_9/2_	0.02	0.22	0.17
^4^F_9/2_	0.59	0.12	0.13
^4^S_3/2_	0.31	0.36	0.34
^2^H_11/2_	0.23	0.62	0.42
^4^F_7/2_	0.98	0.16	0.13

(B) Tm^3+^	
^3^H_6_ →	this work
^3^F_4_	0.14
^3^H_5_	0.66
^3^H_4_	0.55
^3^F_3_	0.87
^3^F_2_	0.28
^1^G_4_	0.03
^1^D_2_	0.24

(C) Yb^3+^		
^2^F_7/2_ →	this work	experiment^32^ ^a^
^2^F_5/2_	1.60	30.72

a The oscillator strengths were multiplied by 10^6^.

b Values are from a sample containing 10 mol % Er^3+^ and 20 mol % Yb^3+^. The transition from the ground state of Er^3+^ to the ^4^I_11/2_ multiplet could therefore not be observed.

Agreement between the calculated values for β-NaYF_4_:Er^3+^ and the experimental data of Zhang et al. for the same dopant–host system is not as good as for LiYF_4_ and its respective reference data (Table 5A).^32^ The trends for β-NaYF_4_:Er^3+^ by the ab initio model and the actual values of the experimental data did not show a good match, although the magnitudes of the values were similar. Remarkably, however, the computed results for β-NaYF_4_:Er^3+^ very closely matched those calculated for LiYF_4_:Er^3+^ (Table 2A), both in trend and actual value. This is down to the fact that the oscillator strength for the ^2^H_11/2_ multiplet is severely underestimated in both data sets. Additionally, crystal field spectra calculated from our ab initio data for β-NaYF_4_:Er^3+^ showed very good agreement for the two multiplets examined (Figure 5A,B, as well as the discussion above). Furthermore, oscillator strengths reported by Villanueva-Delgado et al. for β-NaGdF_4_:Er^3+^ reproduced the trends observed for the multiplet oscillator strengths of LiYF_4_:Er^3+^ and β-NaYF_4_:Er^3+^ predicted through the ab initio calculations.^92^ The same observations were made for the multiplet oscillator strength of Yb^3+^ (Table 5C). The computed values for β-NaYF_4_ and LiYF_4_ were similar and of the same order of magnitude as the experimental results for β-NaGdF_4_. The experimental value reported by Zhang et al. for β-NaYF_4_:Yb^3+^, however, is more than 1 order of magnitude higher.^32^ Reasons for the relatively large disagreement between the ab initio and experimental multiplet oscillator strengths of β-NaYF_4_(:Er^3+^, Tm^3+^, and Yb^3+^) are not clear and require further investigation.

Finally, the multiplet oscillator strengths varied only very slightly from cluster to cluster. This observation is remarkable as it is opposed to the trends observed for the crystal field oscillator strengths, which differed significantly from cluster to cluster. Also, the lack of variation in multiplet oscillator strength suggests that disordered materials do not induce further emission based on stronger oscillator strengths if the available doping sites are already of lowest symmetry (see the file *Oscillator-Strengths_NaYF4-b_Ln* in the RDR for individual values). Therefore, if one is interested in only predicted multiplet oscillator strengths, it might be sufficient to run a single calculation even for a disordered structure, although it is still recommended to pick a configuration of that structure with a high weighting factor. In general, the chosen ab initio approach is well-suited for predicting multiplet oscillator strengths for the materials under consideration.

#### Influence of the 2h Disorder

So far, only the influence of the disorder at the 1f sites onto the optical properties of the Ln^3+^ was examined. However, it was shown previously that also the second disordered site, 2h, has considerable influence on the structure of the host crystal.^29^ Changes at these sites are, in fact, at least as probable as at the 1f sites. This is due to the resulting clusters being generally less unfavorable in terms of total energy compared to the energetic differences of the 1f clusters discussed so far.^29^ It was therefore expected that a similar influence on the optical properties of the Ln^3+^ could be observed.

To obtain first insights into the significance of the 2h disorder, three more embeddings from clusters with the same 1f configuration but different 2h configurations were created and subsequently submitted to the ab initio calculation. While any selection of three configurations out of millions of possible ones only provides an estimate, these configurations were chosen to represent the relevance of all 2h configurations available. More specifically, the first two clusters, i.e., Na1 and Na2, exhibit a balanced distribution of the six Na^+^ at the available 2h sites, where three Na^+^ are shifted along the +*c*-axis and three along the −*c*-axis by equal amounts. Na1 and Na2 differ in exactly which Na^+^ shifts in either direction. Such a distribution is like placing each three Na^+^ and Yb^3+^ at the six 1f disordered sites around a central Ln^3+^. Of the 64 possible configurations (2^6^), 20 configurations should follow such a distribution according to stochastic considerations (Table 3). This makes Na1 and Na2 good representatives of very likely Na^+^ arrangements at the 2h sites. The third configuration, Na3, represents a more extreme case, where all six Na^+^ ions shift along the +*c*-axis. This is similar to having either six Na^+^ or six Yb^3+^ at the six 1f disordered sites (2 of 64 possible configurations). The motivation for this arrangement was to test whether such an imbalanced distribution would result in a severe change in the properties of central Ln^3+^. The individual results of the three clusters are shown in the RDR, files *Energy-Levels_NaYF4-b_Ln*, *Oscillator-Strengths_NaYF4-b_Ln*, and *Spectrum_NaYF4-b_Er-545nm* under the keywords Na1, Na2, and Na3.

Our previous work showed that the disorder at the 2h sites induced similar local distortions of the F^–^ closest to the Ln^3+^ doping site as the 1f disordered sites.^29^ The data reported here confirmed that these changes have a related effect on the crystal field energy levels and oscillator strengths of the Ln^3+^ as well. The ab initio photoluminescence spectra of the individual Na-disordered clusters (Figure S14B) exposed the same features as reported for the Na^+^–Y^3+^ disordered clusters (Figure S14A): transitions were predicted at too high or low energies, gaps were left between transitions, and oscillator strengths were only roughly representing the experimentally observed trends. Interestingly, even for the highly distorted Na^+^ distribution at the 2h sites, i.e., configuration Na3, only changes already present for Na1 and Na2 were observed, meaning that this imbalanced distribution did not invoke any additional changes.

Photoluminescence spectra generated from a model including both the 2h and 1f disorder could possibly show even better agreement with experimental emission spectra. However, accounting for all possible distributions of Na^+^ at the 2h site results in millions of configurations.^29^ To tackle this challenge, a large degree of automatic input generation and data analysis are likely required. Additionally, structural optimization probably must be performed using an iteratively trained machine-learning model instead of a periodic boundary condition DFT calculation for every disordered configuration. Such an approach is beyond the scope of this work but might be attractive for researchers focusing on emerging technologies.

#### Nano versus Bulk

In a recent investigation, Shi et al. demonstrated that photoluminescence properties of highly (>5 mol %) Ln^3+^-doped ultrasmall nanoparticles are predominantly determined by Ln^3+^ that are located at the surface.^93^ This seems to contrast our finding that the emission spectra of UCNPs can be accurately modeled by a bulk model of the nanoparticle material. Yet, the difference in size between the UCNPs studied by Shi et al. and by us should be kept in mind (5 versus 70 nm), having a significant influence on the number of surface versus bulk sites. Indeed, the authors reported a significant change in luminescence behavior when a passivating shell was added to their nanoparticles.^93^ This change was attributed to the Ln^3+^, which previously were situated at the surface, now being in an environment that more closely resembles the bulk part of the particle. Although no shell was added to our nanoparticles, their relatively large size reduces surface effects and, hence, the influence of surface Ln^3+^ on the overall properties. Importantly, UCNPs envisioned for real-life application typically include a passivating (undoped) shell that, among others, increases their luminescence intensity.^18,94^ The work by Shi et al. suggests that such nanoparticles have their Ln^3+^ reside at bulk sites. Our computational results are in line with that, providing very good agreement between experimental and computed spectra using relatively large UCNPs and bulk conditions, respectively. Therefore, we expect that our bulk-based computational strategy will be well-suited to predicting luminescence properties of diverse UCNPs, especially those that exhibit a core/shell architecture.

## Conclusion

Crystal field energy levels (and thereby transition energies) and corresponding crystal field oscillator strengths were predicted for three Ln^3+^, i.e., Er^3+^, Tm^3+^, and Yb^3+^, doped into an ordered (LiYF_4_) and a disordered (β-NaYF_4_) host lattice. This was achieved by using previously geometry optimized supercells of these structures for creating a variety of embedded clusters with Ln^3+^ in the center of the clusters. The three parts of the embedding (quantum cluster, boundary region, and point charge field) were treated with different quantum-chemical precision by the Orca program.

Results obtained for LiYF_4_:Ln^3+^ and β-NaYF_4_:Ln^3+^ were compared to experimental and semiempirical reference data from the literature. Almost all calculated crystal field energy levels showed good agreement when compared with data in the literature, with RMSEs of 67 cm^–1^ or lower. Calculated multiplet oscillator strengths for LiYF_4_:Ln^3+^ were also in close agreement to experimental values, except for the ^2^H_11/2_ multiplet of Er^3+^. For β-NaYF_4_:Ln^3+^, the calculated multiplet oscillator strengths did vary more significantly from a literature value, although our ab initio values followed the same trends as those found in other structures. Most interestingly, a comparison of the ab initio crystal field energy levels and oscillator strengths with experimental values specifically obtained for this study showed particularly good agreement. This was achieved by generating experimental and computational photoluminescence spectra of the same dopant–host combination.

All of this was possible by employing a high-level wave function-based ab initio computational model and, more importantly, by accounting for the disorder of the β-NaYF_4_ host crystal. The disorder exhibited by its lattice was captured by creating several local configurations (clusters) of the crystal structure. Indeed, it was shown that for a disordered structure, if treated as such, the employed computational model can reliably predict the optical properties of Ln^3+^ doped into the crystal. It became apparent that for maximum accuracy of the crystal field energy levels, it is important to account for the results obtained from all the clusters available individually. This includes assigning appropriate weighting factors that represent the probability of occurrence of the specific cluster in a real nanoparticle. It was further demonstrated that the disorder experienced by the host lattice has a considerable influence on the properties of a Ln^3+^-doped crystal. Using only one single configuration of a disordered structure will most likely not result in meaningful results. Accounting for the disorder of β-NaYF_4_, and other disordered structures, should therefore be a top priority for any computational model if an exact understanding of the material is required.

Knowledge of the (crystal field) energy levels and oscillators strengths is crucially important when trying to understand what governs the efficiency of an, for example, upconverting material. Being able to theoretically predict these properties has therefore the potential to foster the design of new, more efficient upconverting dopant–host systems. As such, this study should serve as a significant steppingstone on the path of accurately modeling properties of Ln^3+^ doped into disordered materials or other optically active or magnetic materials showing signs of disorder. Finally, further research is necessary to show to what extent energy transfer, a key requirement for ETU in upconverting particles, is influenced by this type of disorder.

## Supplementary Material



## Data Availability

The following additional material is available in an RDR at 10.6084/m9.figshare.29391644: Env input files for all clusters; Orca output files for all clusters; energy levels for all β-NaYF_4_ clusters; oscillator strengths between multiplets for β-NaYF_4_; Python script for extracting additional energy levels and oscillator strengths from the output files; and ab initio spectra for the 545 and 845 nm transition for all clusters.

## Notes

Energy levels for LiYF_4_ doped with Tm^3+^ and Yb^3+^; additional experimental details; X-ray diffractograms, TEM images, size distributions, and photoluminescence spectroscopy data for all synthesized UCNPs; Env input files for LiYF_4_:Er^3+^ and cluster 10 of β-NaYF_4_:Er^3+^; and Orca input files for cluster 10 and cluster 12 of β-NaYF_4_:Er^3+^ (PDF)

## §

IT Center (ZDV), University of Tübingen, Tübingen (Baden-Württemberg), 72074, Germany

## Abbreviations

AutoAux: autogenerated fitting basis sets
AVAS: atomic valence active space
CASSCF: complete active space self-consistent field
COSX: chain-of-spheres approximation
DFT: density functional theory
ECP: effective core potential
ETU: energy transfer upconversion
Ln^3+^: trivalent lanthanide ion
NEVPT2: N-electron valence state second-order perturbation theory
QDPT: quasi-degenerate perturbation theory
RDR: research data repository
RI: resolution of identity
RMSE: root-mean-square error
RT: room temperature
SA: state-averaged
SOC: spin–orbit coupling
SS: state-specific
TEM: transmission electron microscopy
UCNP: upconverting nanoparticle
XRD: X-ray powder diffraction
