# Supplementary material for: Effect of Disorder on the Emission Spectra of Er3+, Tm3+, and Yb3+-Doped β‑NaYF4: Quantum Chemical and Experimental Results
Source: J Phys Chem C Nanomater Interfaces. 2025 Oct 30;129(45):20446–64. doi: 10.1021/acs.jpcc.5c06209 (PMC12621255; doi:10.1021/acs.jpcc.5c06209)
Supplement: Supplementary file 1 [file jp5c06209_si_001.pdf]

Electronic Supplementary Information (ESI)

Effect of Disorder on the Emission Spectra of Er^{3+}, Tm^{3+}, and Yb^{3+} Doped β-$NaYF_4$: Quantum Chemical and Experimental Results

Chris Steve Conrad,[a,b] Stefan Behnle,[a,†] Eva Hemmer,[b,*] Reinhold F. Fink[a,*]

a Institute of Physical and Theoretical Chemistry, University of Tübingen, Tübingen (Baden-Württemberg), 72076, Germany.

b Department of Chemistry and Biomolecular Sciences, University of Ottawa, Ottawa (Ontario), K1N 6N5, Canada.

† Present Address: IT Center (ZDV), University of Tübingen, Tübingen (Baden-Württemberg), 72074, Germany.

* Corresponding Authors E-mail:

Reinhold.fink@uni-tuebingen.de

ehemmer@uottawa.ca

Table of Contents

1 Crystal Field Energy Levels 3
2 Synthesis and Structural Characterization of Ln^{3+}-doped β-$NaYF_4$ UCNPs 5
2.1 Additional Experimental Details 5
2.2 Preparation of Ligand-Free UCNPs for Photoluminescence Characterization 6
2.3 Structural Characterization 7
3 Optical Characterization of the Synthesized UCNPs 10
4 Env Input Files 17
4.1 $LiYF_4$: Ln^{3+} 17
4.2 β-$NaYF_4$: Ln^{3+} 17
5 Orca Input Files 19
5.1 β-$NaYF_4$: Er^{3+}, Cluster 10, AVAS 19
5.2 β-$NaYF_4$: Er^{3+}, Cluster 12, Mergefrag 21
6 References 26

1 Crystal Field Energy Levels

Table S1: Calculated (*ab initio* and fitted) and experimental crystal field energy levels of $LiYF_4$: Tm^{3+}. Root mean square errors (RMSE) for the *ab initio* results and fitted values compared to the experimental results are reported in the last row.

Crystal Field Energy Level[a] ($^{2S+1}L_J$)		This Work (cm^{-1})	Experiment[1, b] (cm^{-1})	Fitted[2] (cm^{-1})	Crystal Field Energy Level ($^{2S+1}L_J$)		This Work (cm^{-1})	Experiment[1, b] (cm^{-1})	Fitted[2] (cm^{-1})
$^3H_6$	1	0	0	−7	$^3H_4$	1	12 601	12 599	12 595
	2	43	27	24		2	12 632	12 624	12 621
	3	43				3	12 632		
	4	90	56	56		4	12 638	12 643	12 635
	5	298	270	270		5	12 763	12 745	12 745
	6	339	305	298		6	12 770	12 804	12 804
	7	344	319	333		7	12 831	12 835	12 838
	8	363	334	347		8	12 831		
	9	363				9	12 870	12 891	12 914
	10	378	372	387	$^3F_3$	1	14 392	14 520	14 517
	11	379				2	14 392		
	12	385	407	391		3	14 416	14 549	14 544
	13	409	419	417		4	14 468	14 594	14 591
$^3F_4$	1	5 611	5 599	5 600		5	14 472		14 592
	2	5 743	5 756	5 753		6	14 472		
	3	5 743				7	14 478	14597	14 607
	4	5 774	5 757	5 768	$^3F_2$	1	15 018	15 094	15 101
	5	5 795	5 820	5 816		2	15 097	15 203	15 202
	6	5 896	5 942	5 943		3	15 097		
	7	5 903	5 968	5 964		4	15 100		15 231
	8	5 914	5 972	5 969		5	15 157	15 275	15 270
	9	5 914			$^1G_4$	1	21 076	20 973	20 988
$^3H_5$	1	8 269	8 284	8 276		2	21 264	21 186	21 191
	2	8 287	8 300	8 294		3	21 264		
	3	8 292	8 319	8 317		4	21 345	21 272	21 269
	4	8 292				5	21 418	21 300	21 315
	5	8 497	8 501	8 497		6	21 532		21 475
	6	8 504	8 519	8 526		7	21 560	21 554	21 536
	7	8 504				8	21 578	21 562	21 547
	8	8 514		8 539		9	21 578		
	9	8 514		8 542	$^1D_2$	1	28 047	27 961	27 955
	10	8 514				2	28 104	27 991	28 003
	11	8 536	8 535	8 550		3	28 136	28 053	28 055
						4	28 136		
						5	28 197	28 075	28 067
					RMSE		64		9

a: Irreducible representations for all levels are available in the work of Dulick *et al.*[1] b: The concentration of Tm^{3+} in the sample was either 1.5 mol%, 5 mol%, or 7 mol% or the results are an average of all of these samples.

Table S2: Calculated (*ab initio* and fitted) and experimental crystal field energy levels of $LiYF_4$: Yb^{3+}. RMSEs for the *ab initio* results and fitted values compared to the experimental results are reported in the last row.

Crystal Field Energy Level ($^{2S+1}L_J$)		This Work (cm^{-1})	Experi-ment[3, a] (cm^{-1})	Fitted[3] (cm^{-1})
$^2F_{7/2}$	1	0	0	0
	3	271	237	236
	5	357	375	398
	7	462	477	449

Crystal Field Energy Level ($^{2S+1}L_J$)		This Work (cm^{-1})	Experi-ment[3, a] (cm^{-1})	Fitted[3] (cm^{-1})
$^2F_{5/2}$	1	10 196	10 288	10 288
	3	10 354	10 420	10 409
	5	10 538	10 570	10 566
	RMSE	47		14

a: The concentration of Yb^{3+} in the sample was not clearly specified but presumably is 5 mol%.

2 Synthesis and Structural Characterization of Ln^{3+}-doped β-$NaYF_4$ UCNPs

2.1 Additional Experimental Details

Oleate-capped β-$NaYF_4$ UCNPs (co-)doped with Er^{3+}, Tm^{3+}, and Yb^{3+} were synthesized following a synthesis originally published by Rinkel *et al.*[4] In each synthesis, 5 mmol β-$NaYF_4$: Ln^{3+} UCNPs were synthesized by first creating the respective acetate precursors ([$Y(Ac)_3$] and [$Ln(Ac)_3$], Ln = Er^{3+}, Tm^{3+}, and Yb^{3+}). Therefore, the required amounts of the respective oxides (Table S3) were added to 30 mL of a 1 : 2 mixture of water to acetic acid in a 250-mL three-necked round-bottom flask. The slurry was refluxed under rigorous stirring for several hours at 135°C until it became clear. Afterwards, the solvent was evaporated by drying the precursors over night at 65 °C and slow stirring, yielding a fine white powder.

The resulting powder was subsequently combined with 7.5 mmol sodium oleate (1.5:1 Na^{+}-to-Ln^{3+} ion ratio), 50 mL octadecene, and 50 mL oleic acid. The mixture was degassed while stirring under vacuum at 110°C using a heating mantle until a transparent solution formed and no more bubbles emerged. In the next step, the flask was flushed with nitrogen, followed by quickly adding 22.5 mmol ammonium fluoride to the flask (4.5:1 F^{-}-to-Ln^{3+} ion ratio). Thereafter, vacuum was quickly (2 s) applied to the flask, and the flask immediately flushed with nitrogen, for a total of three times. Afterwards, the temperature was increased to 200 °C and the yellow solution was stirred under reflux for 1 h. The employed heating mantle was subsequently removed from the flask for faster cooling, and the flask was allowed to cool to room temperature. This procedure resulted in the formation of ultra-small (sub-5 nm) sodium-deficient sacrificial α-phase $NaYF_4$: Ln^{3+} UCNPs. The UCNPs were isolated by first washing the reaction mixture with two and a half times the volume of ethanol and centrifugation at 9000 RPM for 5 min. The resulting white pellet was then washed with a 1:3 hexane-to-ethanol mixture and centrifuged using the same conditions.

In the second step of the synthesis, all of the sacrificial α-phase $NaYF_4$: Ln^{3+} UCNPs were redispersed in each 10 mL octadecene and oleic acid in a 100-mL three-necked round-bottom flask and degassed at 110°C as described above. Afterwards, the temperature was increased to 300 °C, dissolving the α-phase UCNPs while simultaneously creating the β-phase UCNPs over the course of 3 h under constant stirring. The employed heating mantle was subsequently removed from the flask for faster cooling, and the flask was allowed to cool to room temperature. This procedure resulted in the formation of large (> 60 nm) β-$NaYF_4$ UCNPs. The UCNPs were isolated and washed as described above. After purification, ca. 800 mg of the white-yellow oleate-capped β-$NaYF_4$: Ln^{3+} UCNPs were stored in 5 mL of hexane.

Table S3: Overview for the compositions and dopant concentrations of the six β-$NaYF_4$: Ln^{3+} UCNPs synthesized for this study. Additionally, the molar amounts and masses used to prepare the respective [Y(Ac)$_3$] and [Ln(Ac)$_3$] precursors (Ln = Er^{3+}, Tm^{3+}, and Yb^{3+}) are included in the table.

	Host, β-$NaYF_4$, Y^{3+}		Dopant			Co-Dopant		
Sample Code	Concen-tration [mol%]	Y_2O_3 [mmol]; [mg]	Ln^{3+}	Concen-tration [mol%]	Ln_2O_3 [mmol]; [mg]	Ln^{3+}	Concen-tration [mol%]	Ln_2O_3 [mmol]; [mg]
$^{4.2}Er$	95.8	2.40; 541.7	Er^{3+}	4.2	0.10; 40.0			
$^{2.1}Er$ / $^{2.1}Tm$	95.8	2.49; 563.2	Er^{3+}	2.1	0.05; 20.8	Tm^{3+}	2.1	0.06; 21.1
$^{2.1}Er$ / $^{2.1}Yb$	95.8	2.40; 542.7	Er^{3+}	2.1	0.05; 20.0	Yb^{3+}	2.1	0.05; 20.8
$^{4.2}Tm$	95.8	2.39; 540.6	Tm^{3+}	4.2	0.10; 40.2			
$^{0.5}Tm$ / $^{3.7}Yb$	95.8	2.39; 540.5	Tm^{3+}	0.5	0.01; 4.8	Yb^{3+}	3.7	0.09; 36.8
$^{0.5}Tm$ / $^{20.0}Yb$	79.6	2.02; 456.8	Tm^{3+}	0.5	0.01; 4.9	Yb^{3+}	20.0	0.51; 199.9

2.2 Preparation of Ligand-Free UCNPs for Photoluminescence Characterization

400 mg of the oleate-capped β-$NaYF_4$: Ln^{3+} UCNPs dispersed in 2.5 mL hexane were added to a mixture of 9.5 mL hexane and 24 mL of an aqueous solution of hydrochloric acid (pH = 1.5) in a 150 mL Erlenmeyer flask. The mixture was stirred at room temperature overnight. Subsequently, the bi-phasic system was separated using a separation funnel. The organic phase was discarded. 72 mL of acetone were added to the aqueous phase (three times the initial volume) and well shaken, following centrifugation at 9000 RPM for 5 min. Afterwards, the white ligand-free β-$NaYF_4$: Ln^{3+} UCNPs were stored in 5 mL of water until further characterizations.[5]

2.3 Structural Characterization

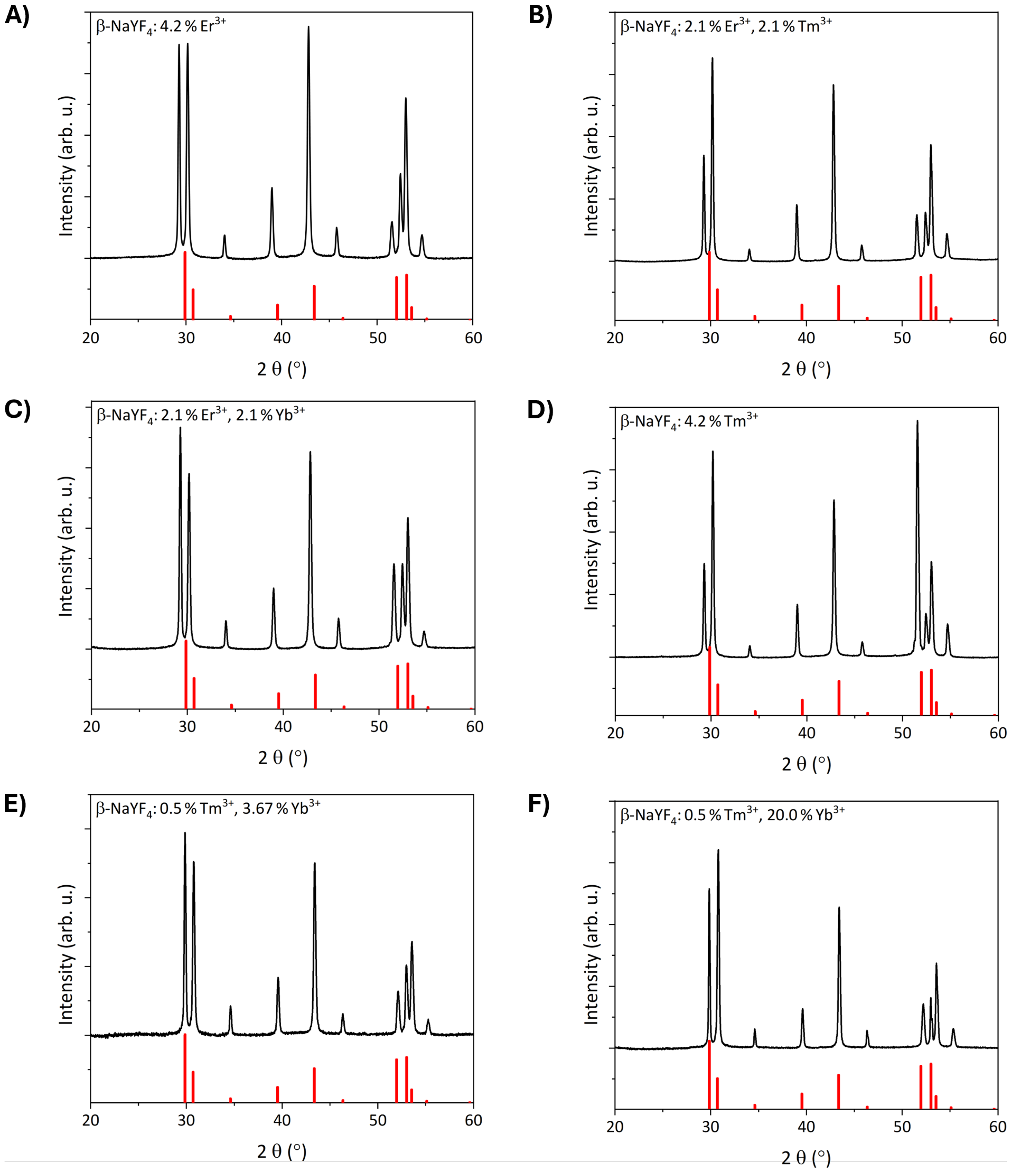

Figure S1: XRD patterns of **(A)** β-NaYF$_4$: 4.2 % Er^{3+}, **(B)** β-NaYF$_4$: 2.1 % Er^{3+}, 2.1 % Tm^{3+}, **(C)** β-NaYF$_4$: 2.1 % Er^{3+}, 2.1 % Yb^{3+}, **(D)** β-NaYF$_4$: 4.2 % Tm^{3+}, **(E)** β-NaYF$_4$: 0.5 % Tm^{3+}, 3.7 % Yb^{3+}, and **(F)** β-NaYF$_4$: 0.5 % Tm^{3+}, 20.0 % Yb^{3+} UCNPs. Reference: β-NaYF$_4$, PDF card [70413].

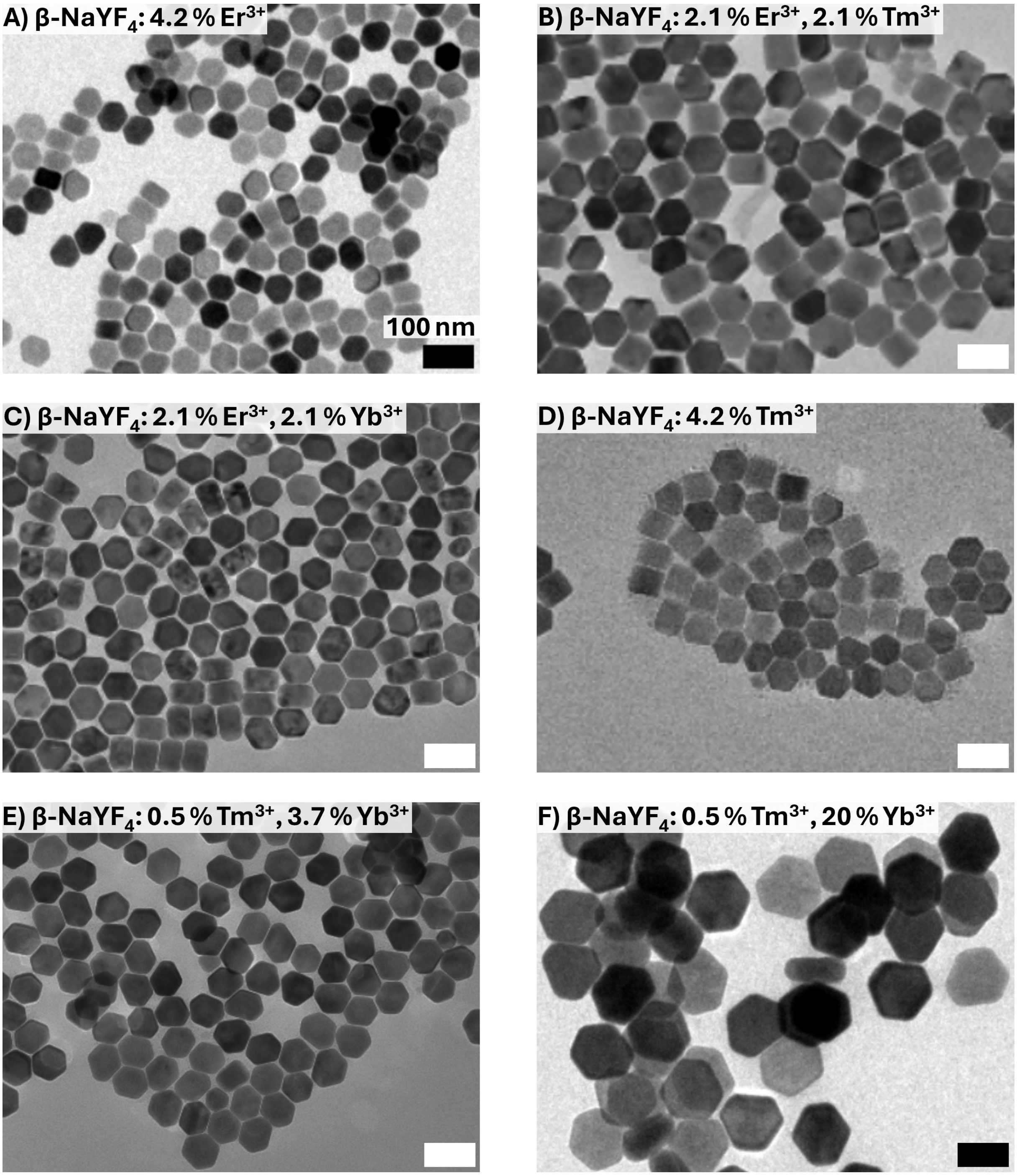

Figure S2: TEM images of **(A)** β-NaYF$_4$: 4.2 % Er^{3+}, **(B)** β-NaYF$_4$: 2.1 % Er^{3+}, 2.1 % Tm^{3+}, **(C)** β-NaYF$_4$: 2.1 % Er^{3+}, 2.1 % Yb^{3+}, **(D)** β-NaYF$_4$: 4.2 % Tm^{3+}, **(E)** β-NaYF$_4$: 0.5 % Tm^{3+}, 3.7 % Yb^{3+}, and **(F)** β-NaYF$_4$: 0.5 % Tm^{3+}, 20.0 % Yb^{3+} UCNPs. The scale bar in (A) applies to all images.

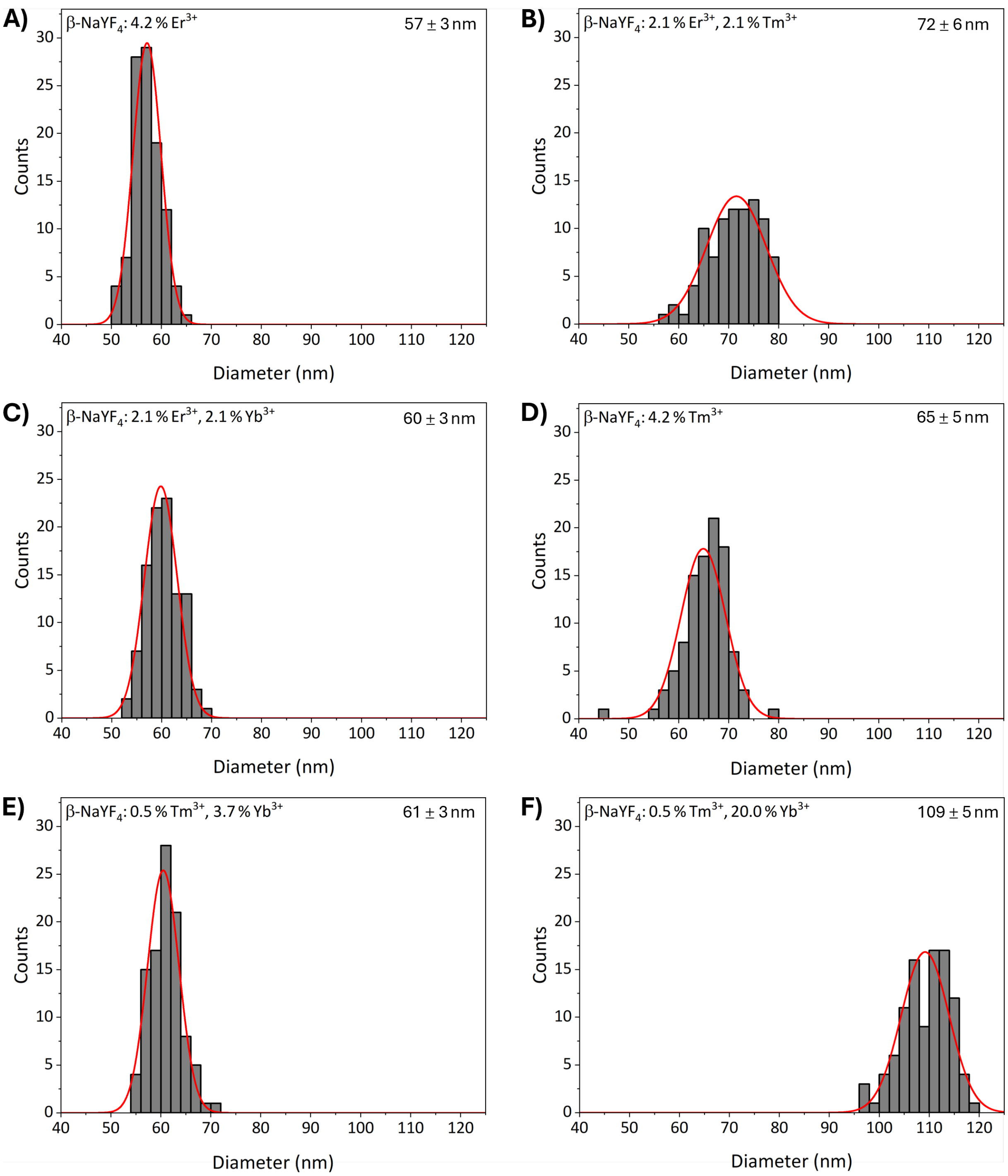

Figure S3: Size distributions of **(A)** β-NaYF$_4$: 4.2 % Er^{3+}, **(B)** β-NaYF$_4$: 2.1 % Er^{3+}, 2.1 % Tm^{3+}, **(C)** β-NaYF$_4$: 2.1 % Er^{3+}, 2.1 % Yb^{3+}, **(D)** β-NaYF$_4$: 4.2 % Tm^{3+}, **(E)** β-NaYF$_4$: 0.5 % Tm^{3+}, 3.7 % Yb^{3+}, and **(F)** β-NaYF$_4$: 0.5 % Tm^{3+}, 20.0 % Yb^{3+}. The diameters of the UCNPs were added to each size distribution in the top right corner.

3 Optical Characterization of the Synthesized UCNPs

Upconversion and Downshifting Emission Spectra, Energy Level Diagrams, Theoretical Spectra

Table S4: Overview of all samples, including color codes, composition, and particle size for the different UCNPs synthesized. Additionally, the temperature, at which the respective photoluminescence spectra were recorded is provided. The applied color code differentiates between the temperatures, *i.e.*, room temperature (RT) and 20 K, at which the photoluminescence spectrum of the respective samples were recorded. It is used consistently in both the manuscript and ESI for all spectra shown.

Sample and Color Code	Composition	Size (nm)	Tempera-ture
$^{4.2}$Er-RT (**magenta**)	β-$NaYF_4$: 4.2 mol% Er^{3+}	57 ± 3	RT
$^{4.2}$Er-Cryo (**olive**)	β-$NaYF_4$: 4.2 mol% Er^{3+}	57 ± 3	20 K
$^{2.1}$Er / $^{2.1}$Tm-Cryo (**orange**)	β-$NaYF_4$: 2.1 % mol% Er, 2.1 mol% Tm^{3+}	72 ± 6	20 K
$^{2.1}$Er / $^{2.1}$Yb-Cryo (**blue**)	β-$NaYF_4$: 2.1 % mol% Er, 2.1 mol% Yb^{3+}	60 ± 3	20 K
$^{4.2}$Tm-RT (**green**)	β-$NaYF_4$: 4.2 mol% Tm^{3+}	65 ± 5	RT
$^{4.2}$Tm-Cryo (**wine**)	β-$NaYF_4$: 4.2 mol% Tm^{3+}	65 ± 5	20 K
$^{0.5}$Tm / $^{3.7}$Yb-Cryo (**brown**)	β-$NaYF_4$: 0.5 % mol% Er, 3.7 mol% Yb^{3+}	61 ± 3	20 K
$^{0.5}$Tm / $^{20.0}$Yb-Cryo (**red**)	β-$NaYF_4$: 0.5 % mol% Er, 20.0 mol% Yb^{3+}	109 ± 5	20 K

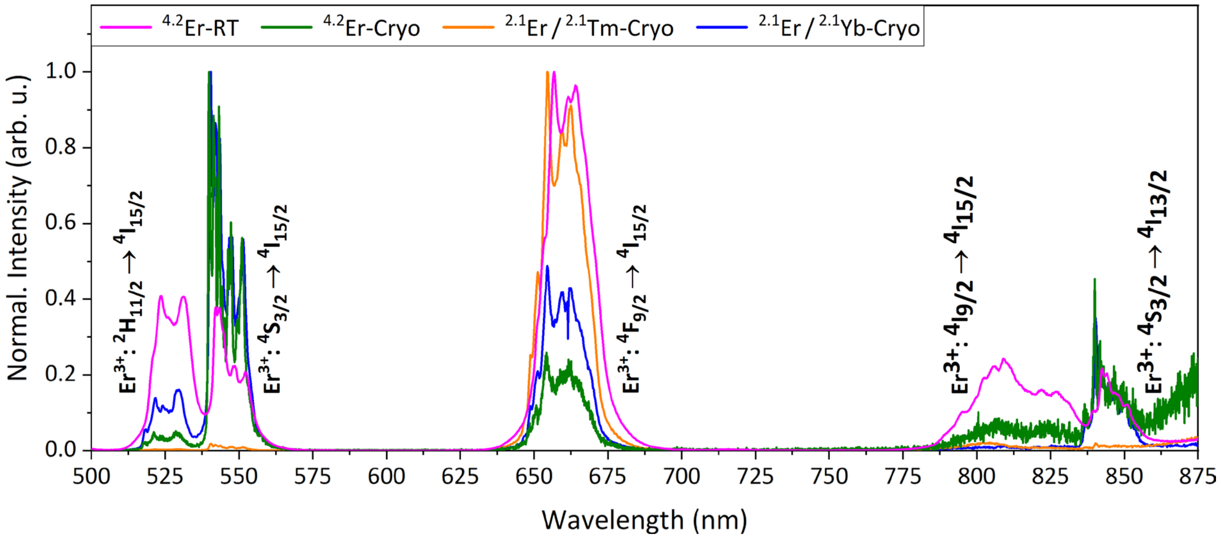

Figure S4: Solid-state upconversion emission spectra of the β-$NaYF_4$ UCNPs doped with 4.2 mol% Er^{3+} recorded at room temperature (color code magenta) and 20 K (color code olive), 2.1 mol% Er^{3+} and 2.1 mol% Tm^{3+} recorded at 20 K (color code orange), as well as 2.1 mol% Er^{3+} and 2.1 mol% Yb^{3+} recorded at 20 K (color code blue). Excitation wavelength (λ_{ex}) = 980 nm; power density = 9.6 W · cm^{-2}.

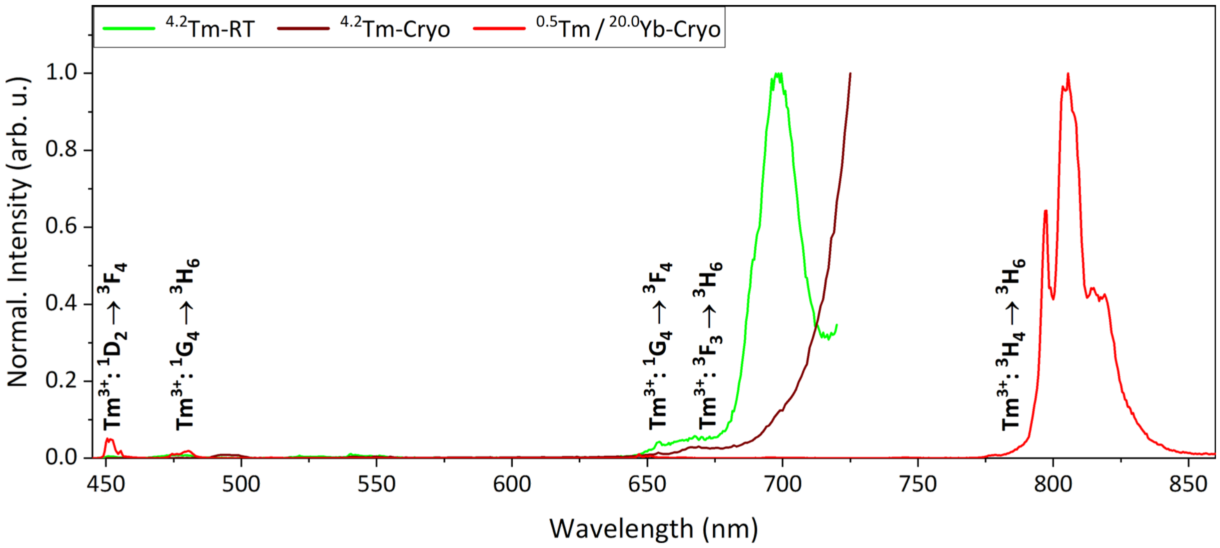

Figure S5: Solid-state upconversion emission spectra of the β-$NaYF_4$ UCNPs doped with 4.2 mol% Tm^{3+} recorded at room temperature (color code green) and 20 K (color code wine), as well as 0.5 mol% Tm^{3+} and 20.0 mol% Tm^{3+} recorded at 20 K (color code red). 4.2 mol% Tm^{3+}: λ_{ex} = 808 nm; power density = 8.1 W · cm^{-2}. 0.5 mol% Tm^{3+} and 20.0 mol% Tm^{3+}: λ_{ex} = 980 nm; power density = 9.6 W · cm^{-2}.

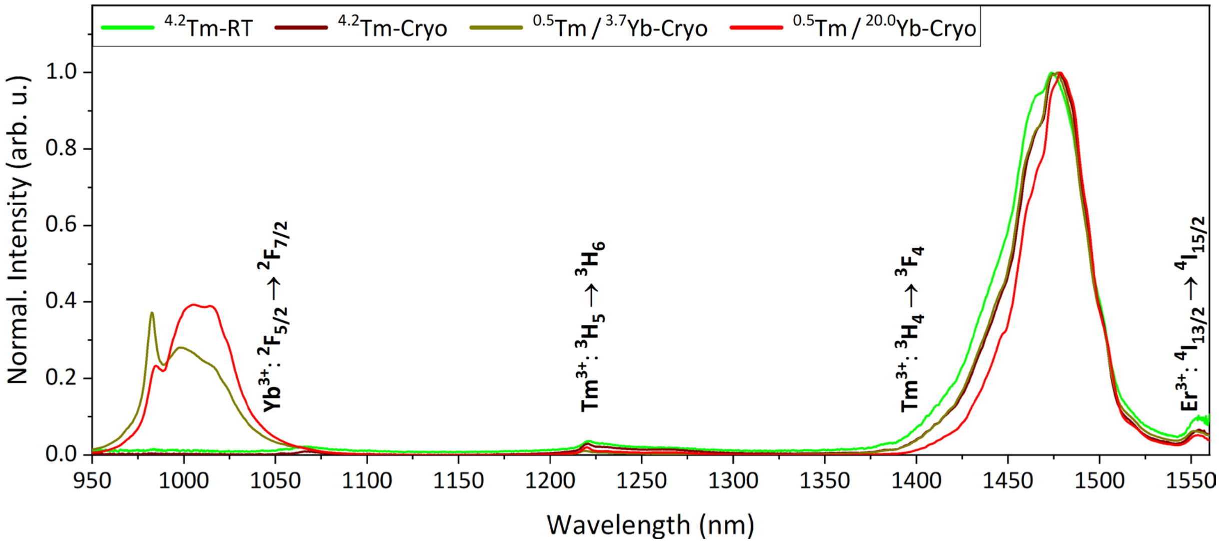

Figure S6: Solid-state downshifting spectra of the β-$NaYF_4$ UCNPs doped with 4.2 mol% Tm^{3+} recorded at room temperature (color code green) and 20 K (color code wine), 0.5 mol% Tm^{3+} and 3.7 mol% Tm^{3+} recorded at 20 K (color code brown), as well as 0.5 mol% Tm^{3+} and 20.0 mol% Tm^{3+} recorded at 20 K (color code red). All samples show contamination with Er^{3+}, which was either already present in the respective oxides or was introduced during the synthesis of the particles. Given the small changes between the differently doped samples, the influence of the Er^{3+} contamination on the positions of the energy levels and oscillator strengths of the corresponding transitions can be considered neglectable. λ_{ex} = 808 nm; power density = 8.1 W · cm^{-2}.

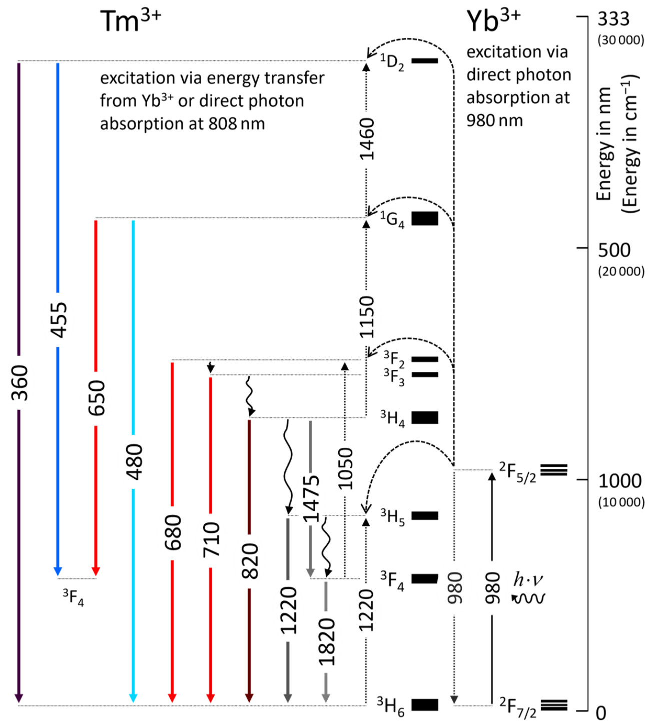

Figure S7: Energy level diagram showing probable upconversion and downshifting pathways for Tm^{3+} together with Yb^{3+} under 980 nm excitation. The photoluminescence spectra of several of the highlighted transitions are presented in Figure S10.

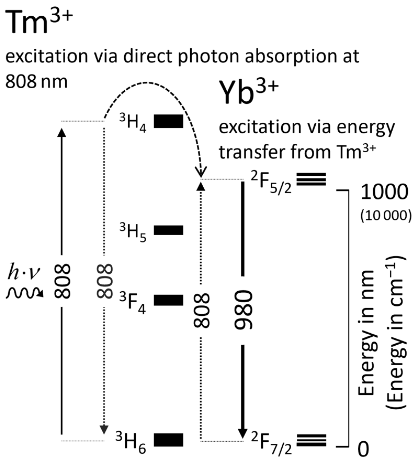

Figure S8: Energy level diagram showing the energy transfer pathways from Tm^{3+} to Yb^{3+} for the photoluminescence spectrum shown in Figure S11.

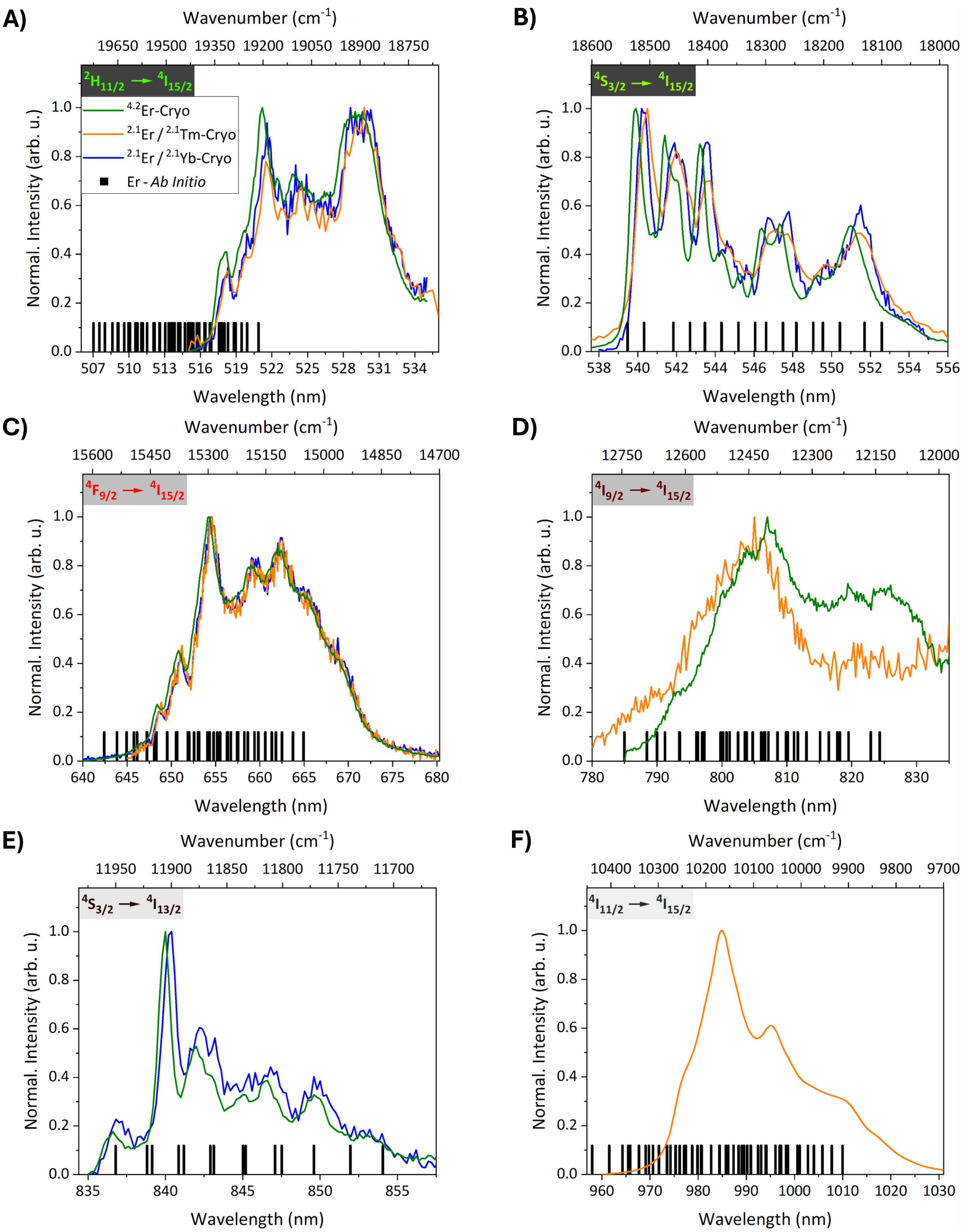

Figure S9: Low-temperature photoluminescence **(A – E)** upconversion and **(F)** downshifting spectra of the β-NaF_4 UCNPs doped with 4.2 mol% Er^{3+}, 2.1 mol% Er^{3+} and 2.1 mol% Tm^{3+}, as well as 2.1 mol% Er^{3+} and 2.1 mol% Yb^{3+} for selected transitions. The legend shown in (A) applies to all spectra. (A – E): $\lambda_{ex} = 980$ nm; power density = 9.6 W · cm^{-2}. (F): $\lambda_{ex} = 808$ nm; power density = 8.1 W · cm^{-2}. Bars in black represent the averaged positions of the crystal field transitions of the respective multiplets according to the *ab initio* calculations. The height of the bars was chosen arbitrarily.

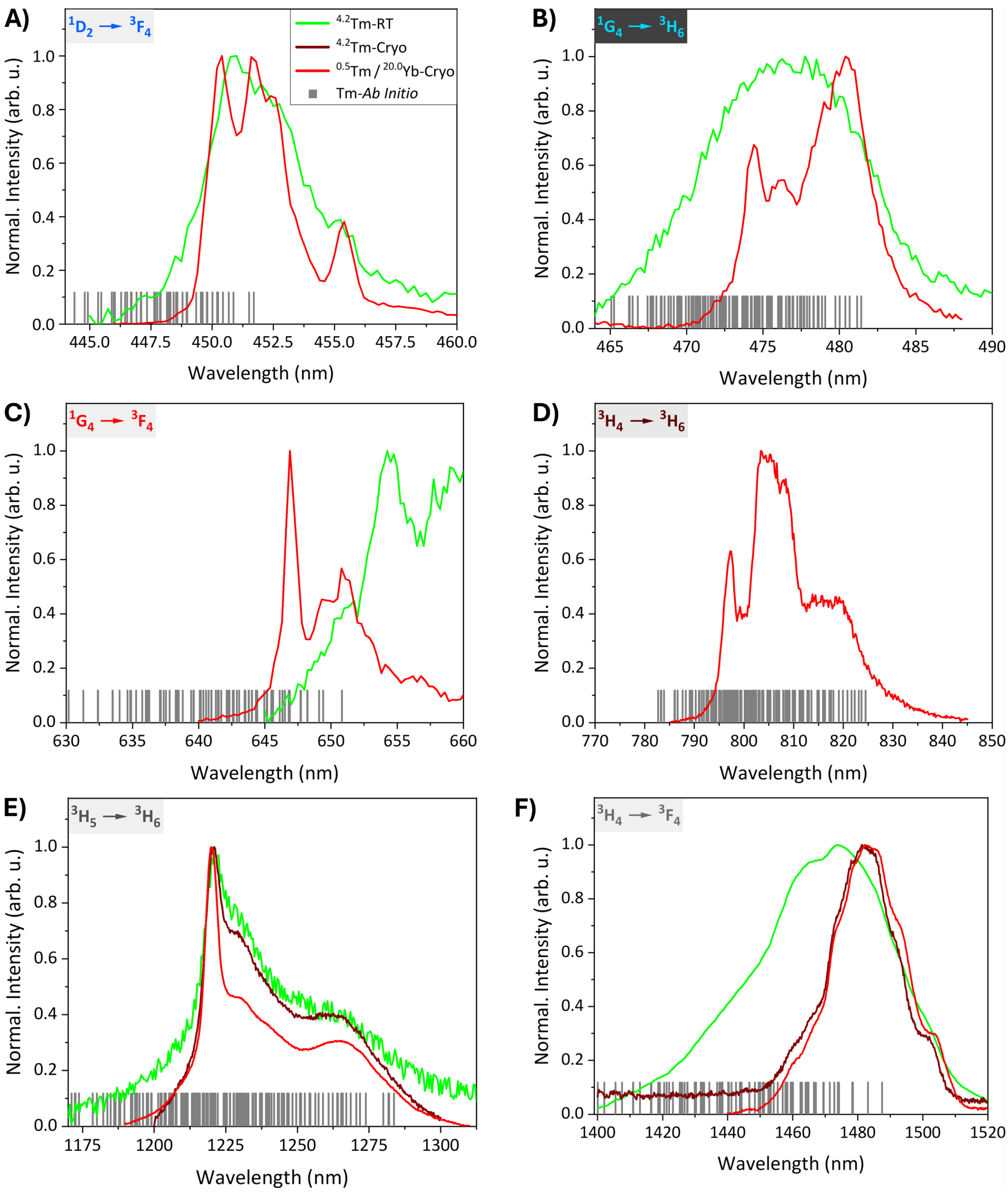

Figure S10: Photoluminescence **(A – D)** upconversion and **(E** and **F)** downshifting spectra of the β-$NaYF_4$ UCNPs doped with 4.2 mol% Tm^{3+} recorded at room temperature and 20 K, as well as 0.5 mol% Tm^{3+} and 20.0 mol% Yb^{3+} recorded at 20 K for specific transitions. The legend shown in (A) applies to the whole figure. 4.2 mol% Tm^{3+}: λ_{ex} = 808 nm; power density = 8.1 W · cm^{-2}. 0.5 mol% Tm^{3+} and 20.0 mol% Tm^{3+}: λ_{ex} = 980 nm; power density = 9.6 W · cm^{-2}. Bars in grey represent the averaged positions of the crystal field transitions of the respective multiplets according to the *ab initio* calculations. The height of the bars was chosen arbitrarily.

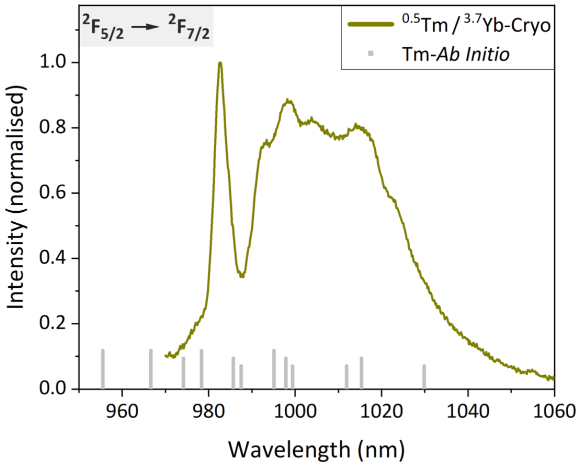

Figure S11: Low-temperature photoluminescence (downshifting) spectrum of β-$NaYF_4$ UCNPs doped with 0.5 mol% Tm^{3+} and 3.7 mol% Yb^{3+} for the 980 nm transitions of Yb^{3+}. λ_{ex} = 808 nm; power density = 8.1 W · cm^{-2}. Bars in light grey represent the averaged positions of the crystal field transitions of the respective multiplets according to the *ab initio* calculations. The different heights of the bars indicate whether a transition originates from the energetically highest (tallest bars), intermediate (medium bars), or lowest (shortest bars) level of the $^2F_{5/2}$ multiplet (they do not represent oscillator strengths).

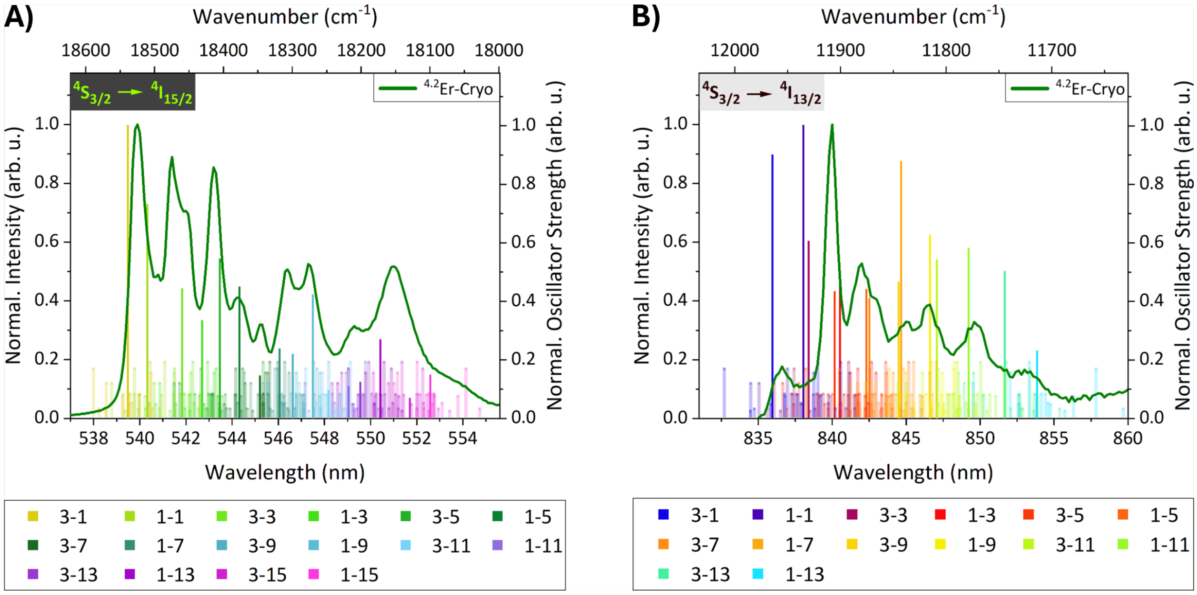

Figure S12: Normalized low-temperature photoluminescence spectra of β-$NaYF_4$ UCNPs doped with 4.2 mol% Er^{3+} (20 K) for the **(A)** $^4S_{3/2} \rightarrow {}^4I_{15/2}$ and **(B)** $^4S_{3/2} \rightarrow {}^4I_{13/2}$ transitions together with the calculated positions of the crystal field transitions and their oscillator strengths. λ_{ex} = 980 nm; power density = 9.6 W · cm^{-2}. The large opaque bars (16 and 14 bars in A and B, respectively) represent the averaged positions of the crystal field transitions of the respective multiplets according to the *ab initio* calculations. Their height is equal to their normalized oscillator strength. Shorter semitransparent bars indicate transitions for the individual clusters (14 clusters with each 16/14 transitions). Their labeling in the legend follows *n^{th} crystal field energy level of the initial multiplet ($^4S_{3/2}$) – n^{th} crystal field energy level of the final multiplet ($^4I_{15/2}$ / $^4I_{13/2}$)*. The height of the smaller bars represents the weighting factor of the cluster they originate from (not their oscillator strength), relative to that of all other clusters. Bars of the same height were retrieved from the same cluster.

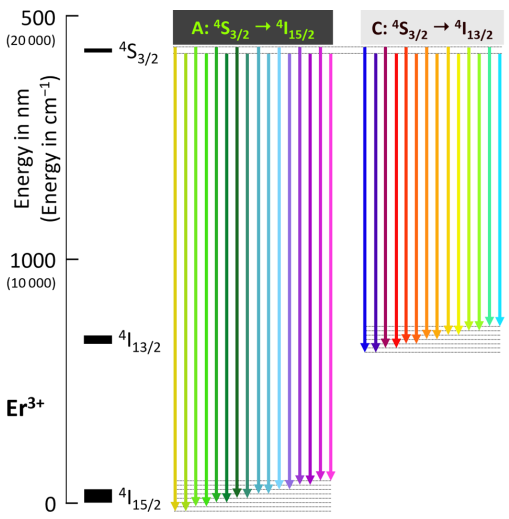

Figure S13: Energy level diagram showing individual crystal field transitions for the $^4S_{3/2} \rightarrow {}^4I_{15/2}$ and $^4S_{3/2} \rightarrow {}^4I_{13/2}$ transitions of Er^{3+}. The separation of the crystal field energy levels is not true to scale.

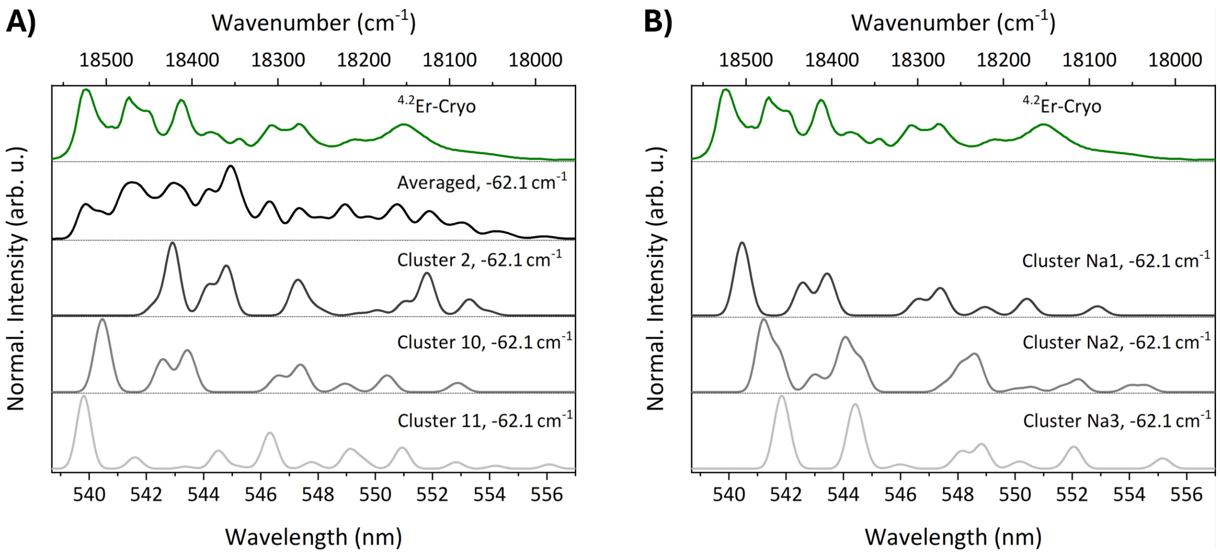

Figure 14: (A) Low-temperature photoluminescence spectrum of Er^{3+}-doped β-$NaYF_4$ for the $^4S_{3/2} \rightarrow {}^4I_{15/2}$ transition (20 K) together with the *ab initio* results for the averaged spectrum. Additionally, individual spectra of cluster 2, cluster 10, and cluster 11 (color code dark grey, grey, and light grey, respectively) are shown. **(B)** Low-temperature photoluminescence spectrum of Er^{3+}-doped β-$NaYF_4$ for the $^4S_{3/2} \rightarrow {}^4I_{15/2}$ transition (20 K). Additionally, individual spectra of cluster Na1, cluster Na2, and cluster Na3 (color code dark grey, grey, and light grey, respectively) are shown. All *ab initio* spectra were shifted by −62 cm^{-1}.

4 Env Input Files

4.1 $LiYF_4$: Ln^{3+}

```
&envin
! output format
Sortie="orca",
! prefix of the generated files and prefix of the coordinate file
Prefix="LiYF4_Er",
NFI_In="LiYF4_Er.cell",
! number of atoms in the .cell file and number of unit cells surrounding the qc
NCh=254,
NCel = 0,
! unit cell lengths and angles
a=15.596216, b=15.597938, c=10.789323,
alpha= 90.008492, beta=89.989532, gamma=89.993942,
! name and fractional coordinates of the central atom in the quantum cluster
Atom0="Er",
Pos0= 0.33317, 0.33237, 0.50056,
! number, names, and fractional coordinates of the magnetic atoms (in the qc)
NMag=1,
AtomMag="Er",
PosMag= 0.33317, 0.33237, 0.50056,
! maximum distance between the central atom and the qc, and between the qc and the ECPs
DSys = 3.0,
DPseud=6.6,
&end
```

4.2 β-$NaYF_4$: Ln^{3+}

```
&envin
! output format
Sortie="orca",
! prefix of the generated files and prefix of the coordinate file
Prefix="10-Er_Na1",
NFI_In="10-Er_Na1.cell",
! number of atoms in the .cell file and number of unit cells surrounding the qc
NCh=144,
```

```
NCel = 0,
! unit cell lengths and angles
a=12.054222, b=12.069157, c=14.151001,
alpha=90.008041, beta=89.996727, gamma=119.791977,
! name and fractional coordinates of the central atom in the quantum cluster
Atom0="Er",
Pos0= 0.498079, 0.498427, 0.499946,
! number, names, and fractional coordinates of the magnetic atoms (in the qc)
NMag=1,
AtomMag="Er",
PosMag= 0.498079, 0.498427, 0.499946,
! maximum distance between the central atom and the qc, and between the qc and the ECPs
DSys = 2.8,
DPseud=7.0,
&end
```

5 Orca Input Files

5.1 β-$NaYF_4$: Er^{3+}, Cluster 10, AVAS

```
# CASSCF+NEVPT2+QDPT calculation of 10-NaYF4_Na1: 4.3 % Er3+ (Er-F9-Na19Y22F62) with 35 quartet and 112 doublet roots

% Pal NProcs 24 end             # number of cores for parallel processing
% MaxCore 6500                  # amount of RAM per core

% PointCharges "pointcharges.pc"          # file that contains all point charges
% Method
  DoEQ true                     # include interactions between point charges
  FrozenCore FC_None            # no frozencore approximation, calculates correlation effects for electrons on low-lying orbitals
end

! AVAS(valence-f)      # determines good (f-) starting orbitals for the active space
% CASSCF
  NEl 11                                  # number of electrons
  NOrb 7                                  # number of orbitals
  Mult 4,2                      # included multiplicities
  NRoots 35,112                           # number of multiplets per multiplicity
  BWeight = 0.238095, 0.761905            # weight per mutliplicity block
  MaxIter 500                   # maximum number of iterations to achieve convergence criteria
  ETol 1e-8                     # energy convergence setting (default is 1e-7)
  GTol 1e-5                     # gradient convergence setting
  ActConstraints 0              # requests natural orbital construction
  trafostep RI                  # utilize the RI-MO algorithm for faster integral transformation
  ActOrbs fOrbs                           # requests AILFT analysis
  PTMethod SC_NEVPT2            # strongly contracted MR approach, provides a DEC corrected total energy
  PTSettings                    # settings for the chosen perturbation theory method
    D3Tpre 1e-15                          # truncation of the third-order reduced density matrix (tight setting)
    D4Tpre 1e-15                          # fourth-order rdm, avoids intruder states (extremely tight setting)
  end
  DoHigherMoments true                    # request the multipole expansion up to 2nd order (magnetic dipole and electric quadrupole contributions), introduces origin-dependence
  Rel                                     # enter into relativistic calculation
    DoSOC true                  # spin-orbit coupling via QDPT
    NInitStates 364             # number of inital SOC states to compute the transition moments from
    Temperature 1000000         # temperature for calculating the Boltzmann distribution
  end
end

% Rel                                     # include relativistic effects
  Method DKH                    # (relativistic) Hamiltonian operator
  Order 2                       # second-order DKH Hamiltonian
  PictureChange 2               # include the second-order DKH transformation of the SOC operator and the first order of the Zeeman operator
  FiniteNuc true                          # account for the spatial extent of the nuclei (opposed to the point charges used for the elementary particles)
end

! AutoAux                       # automatic construction of a auxiliary basis set for Coulomb-, exchange-, and correlation-fitting
% Basis
  Basis "DKH-DEF2-TZVPP"# general basis set
  NewGTO
    Er "SARC2-DKH-QZVP" end
  NewECP                        # ECP per element
    Na "SDD" end
  NewECP                        # ECP per element
    Y "SDD" end
end

* xyz -6 4                      # keyword, total charge, multiplicity, (file name)
  Er     0.000000000000     0.000000000000     0.000000000000
  f     -2.288293866532     0.264219131682     0.154104399356
  f     -0.764422676182    -1.236186817617     1.778695901990
  f     -0.890452277930    -1.245531670436    -1.777974200946
  f      0.901031047951    -2.118480423942    -0.149646834086
  f     -0.652430405736     1.453640812048     1.774139279713
  f     -0.685848639863     1.321738560667    -1.777195895899
  f      1.496152743901    -0.032761847666     1.778837411999
  f      1.576211860870    -0.061662684727    -1.770417566487
  f      1.359225926221     1.956778621591    -0.147085502930
  na>     1.000000000000     -5.837462991042    3.508160738213   -1.601765938248
   y>     3.000000000000     -6.017756529567    0.028568661062   -3.537283231759
   y>     3.000000000000      6.008547470344    0.002211016831   -0.007712295468
   y>     3.000000000000     -6.020682748207    0.022229583649    3.543764390152
   y>     3.000000000000     -2.988002297169    1.787849206282   -5.310997981446
  na>     1.000000000000     -2.994288366143    1.761756782630   -1.888734084671
   y>     3.000000000000     -3.013599305164    1.801549085398    1.753592026466
  na>     1.000000000000     -3.049130920577    1.761412214588    5.328644279518
   y>     3.000000000000     -3.013670802338   -5.206731368023   -3.544047410170
   y>     3.000000000000     -3.008972446713   -5.196997612085    0.002051895125
   y>     3.000000000000     -3.039232045980   -5.212022481241    3.532655854478
  na>     1.000000000000     -2.892644931270   -1.728342792114   -5.269832719947
  na>     1.000000000000     -3.117200553127   -1.730281064809   -1.774294940723
  na>     1.000000000000     -2.946536963787   -1.728198405309    1.892130324877
  na>     1.000000000000     -3.116082550569   -1.731787125482    5.271983672078
  na>     1.000000000000     -0.023713871479   -3.521825753021   -5.327130122426
   y>     3.000000000000     -0.007235836938   -3.451501785362   -1.764799619146
  na>     1.000000000000      0.003598316679   -3.474697776469    1.776445892853
   y>     3.000000000000      0.009563166295   -3.445531132356    5.295035652477
```

```
y>   3.000000000000   0.029896818201   0.033492219871  -3.543184199117
y>   3.000000000000   0.000104553872   0.059277885641   3.531113395384
na>  1.000000000000   0.188263952816   3.509323652404  -5.501074224987
na>  1.000000000000  -0.034288238537   3.506912933477  -1.834521600379
na>  1.000000000000   0.137113199849   3.507251995543   1.545190236813
na>  1.000000000000  -0.088196485810   3.505632873118   5.327271632435
na>  1.000000000000   3.032245866219   1.761980419041  -5.327158424428
y>   3.000000000000   3.041283552673   1.781693990052  -1.766002454219
na>  1.000000000000   3.031231160018   1.760767554736   1.719714530409
y>   3.000000000000   3.015333236939   1.781356400910   5.316092341756
y>   3.000000000000   3.023605951204  -5.209538793396  -3.541075699989
y>   3.000000000000   3.042402805652  -5.180467680774  -0.012679296770
na>  1.000000000000   2.909371934939  -1.729649248073  -5.616277522981
na>  1.000000000000   3.080843641767  -1.729076664134  -2.179353189315
na>  1.000000000000   2.909180898898  -1.729817205698   2.180640930393
na>  1.000000000000   3.187849876032  -1.729344891637   5.501725171026
f>  -1.000000000000  -3.671122164266  -3.806556555007   1.771648703562  NewECP "SDD" end
f>  -1.000000000000  -3.741142530187  -3.932132849339  -1.776884573880  NewECP "SDD" end
f>  -1.000000000000  -4.459125978031  -0.071130010625  -5.270823290007  NewECP "SDD" end
f>  -1.000000000000  -4.479772716203  -0.031504174524   1.772724179627  NewECP "SDD" end
f>  -1.000000000000  -4.533999961350  -0.032250823088  -1.776346835847  NewECP "SDD" end
f>  -1.000000000000  -4.509801361264  -0.030866936721   5.276809163371  NewECP "SDD" end
f>  -1.000000000000  -4.720139015370   1.957568172472   0.146675123905  NewECP "SDD" end
f>  -1.000000000000  -4.669173430926   1.954900683893  -3.695434817361  NewECP "SDD" end
f>  -1.000000000000  -4.715982371792   1.958824134757   3.401334566505  NewECP "SDD" end
f>  -1.000000000000  -1.686113669656  -3.312416235594  -0.147311918944  NewECP "SDD" end
f>  -1.000000000000  -1.673889905133  -3.285140832677  -3.402650609585  NewECP "SDD" end
f>  -1.000000000000  -1.681448470011  -3.282715545223   3.697033880458  NewECP "SDD" end
f>  -1.000000000000   0.714516538470  -5.021332569770  -0.198793260069  NewECP "SDD" end
f>  -1.000000000000   0.714304612173  -5.013290638932  -3.348607937304  NewECP "SDD" end
f>  -1.000000000000   0.699328686183  -4.978095674774   3.697684826497  NewECP "SDD" end
f>  -1.000000000000  -2.294678001002   0.261228780870  -3.702213146772  NewECP "SDD" end
f>  -1.000000000000  -2.308427069337   0.258150794901   3.350419265414  NewECP "SDD" end
f>  -1.000000000000  -0.718643384081  -1.250840436581  -5.281224275639  NewECP "SDD" end
f>  -1.000000000000  -0.880974834064  -1.250270987785   5.324526338268  NewECP "SDD" end
f>  -1.000000000000  -3.888277086848   3.987656210268   1.773092105650  NewECP "SDD" end
f>  -1.000000000000  -3.701718139137   3.994167981914  -1.775299661784  NewECP "SDD" end
f>  -1.000000000000  -2.016527058264   3.163527114593   0.152590242264  NewECP "SDD" end
f>  -1.000000000000  -2.015541980621   3.159876948724  -3.704109380888  NewECP "SDD" end
f>  -1.000000000000  -2.052866861503   3.127569580027   3.401377019508  NewECP "SDD" end
f>  -1.000000000000   0.987428693650  -2.082539497500  -3.350886248442  NewECP "SDD" end
f>  -1.000000000000   0.983301769528  -2.065978420748   3.699864080630  NewECP "SDD" end
f>  -1.000000000000  -0.634719383650   1.437588079137  -5.319941413989  NewECP "SDD" end
f>  -1.000000000000  -0.731038656733   1.320560160409   5.327285783435  NewECP "SDD" end
f>  -1.000000000000   2.277219613995  -3.915168901290  -5.318200840884  NewECP "SDD" end
f>  -1.000000000000   2.316825634506  -3.915958595597   1.777238348901  NewECP "SDD" end
f>  -1.000000000000   2.370831738236  -3.801343861560  -1.774436450731  NewECP "SDD" end
f>  -1.000000000000   2.364434482778  -3.792334083728   5.272578014114  NewECP "SDD" end
f>  -1.000000000000  -1.427308003719   5.157280769980   1.778596844984  NewECP "SDD" end
f>  -1.000000000000  -1.461950364017   5.205281689379  -1.781313837149  NewECP "SDD" end
f>  -1.000000000000   1.510954696010  -0.035542885439  -5.323691429217  NewECP "SDD" end
f>  -1.000000000000   1.547189850966  -0.036738796403   5.326408421382  NewECP "SDD" end
f>  -1.000000000000   1.383495211951   1.921115731612  -3.403952501664  NewECP "SDD" end
f>  -1.000000000000   1.356269752863   1.948122993079   3.700316912657  NewECP "SDD" end
f>  -1.000000000000   4.413783044986  -3.352538011469   0.144410963768  NewECP "SDD" end
f>  -1.000000000000   4.353005806372  -3.282861225918  -3.698463131545  NewECP "SDD" end
f>  -1.000000000000   4.380938316307  -3.320719732142   3.406230812802  NewECP "SDD" end
f>  -1.000000000000   3.730221715937   0.263755855370  -3.399410030388  NewECP "SDD" end
f>  -1.000000000000   3.721598247504   0.205780860823   3.754402037941  NewECP "SDD" end
f>  -1.000000000000   3.742965951157   0.314524088923  -0.156354408493  NewECP "SDD" end
f>  -1.000000000000   2.308756001980   3.989646330295   1.772681726625  NewECP "SDD" end
f>  -1.000000000000   2.170781578588   3.965898120777  -1.771931723579  NewECP "SDD" end
f>  -1.000000000000   2.141776332332   3.988329370788   5.323479164204  NewECP "SDD" end
f>  -1.000000000000   5.254902312094  -1.242169405883  -1.778738354992  NewECP "SDD" end
f>  -1.000000000000   5.124110525315  -1.278355003289   1.776460043854  NewECP "SDD" end
f>  -1.000000000000   4.023370854432   3.159465412457  -3.355725890736  NewECP "SDD" end
f>  -1.000000000000   3.992931913190   3.125048860464   3.698816906566  NewECP "SDD" end
f>  -1.000000000000   4.062441331257   3.171578585900  -0.148415697011  NewECP "SDD" end
y>   2.999996911163  -6.045674529656   0.002211016831  -0.007712295468
f>  -0.999786768100  -5.067905149161  -2.110104140388  -3.699071624582  NewECP "SDD" end
f>  -0.999867724887  -5.035074970614  -2.075307686216   3.351070211453  NewECP "SDD" end
f>  -0.999854341688  -5.044277648056  -2.076047081163   0.151712880211  NewECP "SDD" end
f>  -0.999997610702  -1.525679720261  -5.260144578026   1.780394022093  NewECP "SDD" end
f>  -0.999991545728  -1.470998509527  -5.279858780703  -1.775865701818  NewECP "SDD" end
y>   2.999956276198  -2.954187357655   5.293569606919  -0.012679296770
y>   2.999998520926  -2.967727962951   5.246962349714   3.542462498073
f>  -0.999789546872   0.752179342796   5.451884062537   0.154486476379  NewECP "SDD" end
f>  -0.999700144440   0.732075452080   5.493996898911  -3.717199056682  NewECP "SDD" end
f>  -0.999695744888   0.731212999469   5.494959800998   3.351636251488  NewECP "SDD" end
y>   2.999993207424   6.036465470433   0.028568661062  -3.537283231759
y>   2.999996188746   6.033539251793   0.022229583649   3.543764390152
f>  -0.999998859950   5.304919691119   1.293167179430   1.781172327140  NewECP "SDD" end
f>  -0.999877882808   5.393631972057   1.435570770336  -1.780082700074  NewECP "SDD" end
y>   2.999959228263   3.048659389980   5.277039675608   0.002051895125
*
```

5.2 β-$NaYF_4$: Er^{3+}, Cluster 12, Mergefrag

Initial Guess for Er^{3+}:

```
# CASSCF calculation with one interation of Er3+ with 35 quartet and 112 doublet roots for initial guess orbitals

% Pal NProcs 24 end          # number of cores for parallel processing
% MaxCore 6500               # amount of RAM per core

% CASSCF
  MaxIter 1                  # determines the number of CASSCF (macro) iterations
  NEl 11                             # number of electrons
  NOrb 7                             # number of orbitals
  Mult 4,2                   # included multiplicities
  NRoots 35,112                      # number of multiplets per multiplicity
  BWeight = 0.238095, 0.761905       # weight per mutliplicity block
  PrintWF true
end

% Method
  FrozenCore FC_None         # no frozencore approximation, calculates correlation effects for electrons on low-lying orbitals
end

% Rel                                # include relativistic effects
  Method DKH                 # (relativistic) Hamiltonian operator
  Order 2                    # second-order DKH Hamiltonian
  PictureChange 2            # include the second-order DKH transformation of the SOC operator and the first order of the Zeeman operator
  FiniteNuc true                     # account for the spatial extent of the nuclei (opposed to the point charges used for the elementary particles)
end

% Output
  Print[p_basis] 2           # prints the basis in the input format
end

! AutoAux                    # automatic construction of a auxiliary basis set for Coulomb-, exchange-, and correlation-fitting
% Basis
  Basis "DKH-DEF2-TZVPP"     # general basis set
  NewGTO                     # basis per element
   Er "SARC2-DKH-QZVP" end
end

* xyz +3 4                   # keyword ; total charge ; multiplicity ; (file name)
  Er     0.0     0.0     0.0
*
```

Full Calculation for Er^{3+} after Restart:

```
# CASSCF+NEVPT2+QDPT calculation of Er3+ with 35 quartet and 112 doublet roots

% Pal NProcs 24 end          # number of cores for parallel processing
% MaxCore 6500               # amount of RAM per core

% SCF
  Guess MORead               # restart from an earlier calculation
  MOInp "input-IG.gbw"       # initial orbitals from a HF calculation
  Convergence verytight      # target precision of the energy and the wavefunction
  Rotate                             # adjust active orbitals from an initial guess
   {27,36}                   # {remove, add}
   {28,37}
   {29,38}
   {30,39}
   {31,40}
   {32,41}
   {33,42}
  end
end

% Method
  FrozenCore FC_None         # no frozencore approximation, calculates correlation effects for electrons on low-lying orbitals
end

% CASSCF
  NEl 11                             # number of electrons
  NOrb 7                             # number of orbitals
  Mult 4,2                   # included multiplicities
  NRoots 35,112                      # number of multiplets per multiplicity
  BWeight = 0.238095, 0.761905       # weight per mutliplicity block
  MaxIter 500                # maximum number of interations to achieve convergence criteria
  ActConstraints 0
  trafostep ri               # utilize the RI-MO algorithm for faster integral transformation
  PrintWF true
  PTMethod SC_NEVPT2         # strongly contracted MR approach, provides a DEC corrected total energy
  PTSettings                 # settings for the chosen perturbation theory method
   D4Tpre 1e-14                      # avoids intruder states (very tight setting)
  end
  DoHigherMoments true       # request the multipole expansion up to 2nd order (magnetic dipole and electric quadrupole contributions), introduces origin-dependence
  rel                                # enter into relativistic calculation
   DoSOC true                # spin-orbit coupling via QDPT
   NInitStates 364           # number of inital SOC states to compute the transition moments from
   Temperature 1000000       # temperature for calculating the Boltzmann distribution
  end
end
```

```
! AutoAux                    # automatic construction of a auxiliary basis set for Coulomb-, exchange-, and correlation-fitting
% Basis                                 # basis per element
  Basis "DKH-DEF2-TZVPP"     # general basis set
  NewGTO                     # basis per element
   Er "SARC2-DKH-QZVP" end
end

*xyz +3 4                    # keyword, total charge, multiplicity, (file name)
  Er     0.0      0.0      0.0
 *
```

DFT Calculation for Surrounding Ions and Point Charges:

```
# DFT calculation of 9 F-, ECPs, and point charges

% Pal NProcs 24 end          # number of cores for parallel processing
% MaxCore 3000               # amount of RAM per core

% SCF
  MaxIter 500                # maximum number of SCF iterations to reach convergence
  Convergence verytight      # target precision of the energy and the wavefunction
end
! PBE0                                  # DFT functional

% PointCharges "pointcharges.pc"        # file that contains all point charges
% Method
  DoEQ true                  # include interactions between point charges
  FrozenCore FC_None         # no frozencore approximation, calculates correlation effects for electrons on low-lying orbitals
end

! AutoAux                    # automatic construction of an auxiliary basis set for Coulomb-, exchange-, and correlation-fitting
% Basis
  Basis "DKH-DEF2-TZVPP"     # general basis set
  NewECP                     # ECP per element
   Na "SDD" end
  NewECP                     # ECP per element
   Y "SDD" end
end

* xyz -9 1                   # keyword, total charge, multiplicity, (file name)
  f     -2.299492273536     0.308637372811    -0.153181543199
  f     -0.818879087031    -1.217957239365    -1.788458596217
  f     -0.749118336035    -1.180309559734     1.766083923179
  f      0.957298398847    -2.089301717118    -0.156636872453
  f     -0.624558199710     1.385730558004     1.705105858557
  f     -0.633420712704     1.448685779723    -1.731034989147
  f      1.620272740359    -0.048917365162     1.708122191061
  f      1.585444746148     0.027856445483    -1.732012110945
  f      1.501069922024     1.966620981619     0.015364886232
   f>    -1.000000000000    -3.616969752303   -3.742219477545    1.756964119738   NewECP "SDD" end
   f>    -1.000000000000    -1.483281727066   -5.213510369862    1.711011072897   NewECP "SDD" end
   f>    -1.000000000000    -4.554457480747    0.073984742263    1.759442327195   NewECP "SDD" end
   f>    -1.000000000000    -4.471358951069   -0.056232986376   -1.780330075759   NewECP "SDD" end
   f>    -1.000000000000    -4.503300747424    0.037250296028   -5.290944597836   NewECP "SDD" end
   f>    -1.000000000000    -4.431057887467   -0.071949672423    5.268853148507   NewECP "SDD" end
   f>    -1.000000000000    -4.701662530124    1.976313126456   -0.165728353523   NewECP "SDD" end
   f>    -1.000000000000    -1.653017848711   -3.247327998787   -3.360760857462   NewECP "SDD" end
   f>    -1.000000000000    -4.676890702508    1.985495062150   -3.357234722281   NewECP "SDD" end
   f>    -1.000000000000    -4.663113037556    1.969747961160    3.690886413075   NewECP "SDD" end
   f>    -1.000000000000    -1.617248029872   -3.254848963274   -0.164666264613   NewECP "SDD" end
   f>    -1.000000000000    -1.638595671890   -3.230301453121    3.641605487649   NewECP "SDD" end
   f>    -1.000000000000     0.749223038664   -5.006143580048   -0.210704278567   NewECP "SDD" end
   f>    -1.000000000000    -2.310979368770    0.280778569443   -3.360732535091   NewECP "SDD" end
   f>    -1.000000000000    -2.295118581786    0.275918003966    3.627500946924   NewECP "SDD" end
   f>    -1.000000000000     0.772107127869   -4.956249997758   -3.358395939489   NewECP "SDD" end
   f>    -1.000000000000     0.766493418574   -4.915732239621    3.689555261641   NewECP "SDD" end
   f>    -1.000000000000    -0.817733798160   -1.228162188904    5.269065566289   NewECP "SDD" end
   f>    -1.000000000000    -3.816450895798    4.008431336318   -1.781094779774   NewECP "SDD" end
   f>    -1.000000000000    -0.798563277331   -1.172839559706   -5.290052443151   NewECP "SDD" end
   f>    -1.000000000000    -3.833265813567    4.052240491136    1.759286554155   NewECP "SDD" end
   f>    -1.000000000000    -2.058737219729    3.188307177482    3.683012793955   NewECP "SDD" end
   f>    -1.000000000000     1.004145483932   -2.038644391723   -3.369781532605   NewECP "SDD" end
   f>    -1.000000000000    -1.996971619225    3.185009744230   -3.361568045034   NewECP "SDD" end
   f>    -1.000000000000     1.033751495854   -2.015588605525    3.636394171397   NewECP "SDD" end
   f>    -1.000000000000    -1.994431917776    3.209551431044   -0.157231642243   NewECP "SDD" end
   f>    -1.000000000000    -0.711936165766    1.396138800890   -5.235333622506   NewECP "SDD" end
   f>    -1.000000000000     2.340946409013   -3.876635334905    1.764257130254   NewECP "SDD" end
   f>    -1.000000000000     2.378521906020   -3.840575335991   -5.288508873935   NewECP "SDD" end
   f>    -1.000000000000    -0.688728740098    1.483251201001    5.209815166294   NewECP "SDD" end
   f>    -1.000000000000     2.407802943759   -3.744709456566   -1.783884533311   NewECP "SDD" end
   f>    -1.000000000000     2.433436540495   -3.706981847047    5.268442474128   NewECP "SDD" end
   f>    -1.000000000000     1.517090797101   -0.036550616726   -5.287517590953   NewECP "SDD" end
   f>    -1.000000000000     1.479107813772    0.069602739607    5.268428312943   NewECP "SDD" end
   f>    -1.000000000000    -1.475470844758    5.167587296065    1.767330107500   NewECP "SDD" end
   f>    -1.000000000000     4.437201106207   -3.274037525842   -3.712864572920   NewECP "SDD" end
   f>    -1.000000000000     1.392532127605    1.976574173895   -3.415366388624   NewECP "SDD" end
   f>    -1.000000000000     4.429250373208   -3.269262433015    3.341855674863   NewECP "SDD" end
   f>    -1.000000000000     1.386101387971    1.972220299282    3.336516907942   NewECP "SDD" end
   f>    -1.000000000000     4.460341376552   -3.303762316061    0.190382977422   NewECP "SDD" end
   f>    -1.000000000000     3.725996229721    0.283955256882   -3.419345681740   NewECP "SDD" end
   f>    -1.000000000000     3.724910305357    0.266818222262    3.393359906408   NewECP "SDD" end
   f>    -1.000000000000     3.738996653948    0.461249658737    0.012603455066   NewECP "SDD" end
   f>    -1.000000000000     2.190011989058    3.967955283430   -1.730865054922   NewECP "SDD" end
```

```
 f>    -1.000000000000     5.184453948238   -1.186394384778    1.754613362950   NewECP "SDD" end
 f>    -1.000000000000     2.243909262942    3.966018355847    1.706819361998   NewECP "SDD" end
 f>    -1.000000000000     5.250111275293   -1.170458331321   -1.778135092012   NewECP "SDD" end
 f>    -1.000000000000     4.011660262190    3.140229669000   -0.039453062712   NewECP "SDD" end
 f>    -1.000000000000     4.035485396296    3.187065114589    3.391009149620   NewECP "SDD" end
 f>    -1.000000000000     4.046317139562    3.192080455337   -3.362587650387   NewECP "SDD" end
 f>    -1.000000000000    -3.722048656733   -3.924665774858   -1.786773415147   NewECP "SDD" end
 na>    1.000000000000    -3.062343860357   -1.804378518666   -2.076964427742
 na>    1.000000000000    -3.069555754538   -1.812246470978    5.321858465711
 na>    1.000000000000    -2.947545973451   -1.606851014237   -5.287503429767
 na>    1.000000000000    -2.904136620751   -1.519840691063    1.882248127567
 na>    1.000000000000     0.042149882197   -3.410402411246    1.823422563136
 na>    1.000000000000    -2.991025551500    1.833991004062   -5.400453045054
 na>    1.000000000000    -2.999516923814    1.828112942700    1.766310502146
 na>    1.000000000000     0.079003550537   -3.354858013524   -5.343411789992
 na>    1.000000000000     2.996384747963   -1.763228909509    2.170187511673
 na>    1.000000000000    -0.015930357109    3.523022365965    1.652086380167
 na>    1.000000000000     3.060192958885   -1.656433202894   -5.519987611583
 na>    1.000000000000    -0.041059624125    3.674240626765   -1.674758438101
 na>    1.000000000000     0.105476993763    3.435442983838   -5.458712162066
 na>    1.000000000000     3.064271237285   -1.662608618084   -2.306970402101
 na>    1.000000000000     0.036136765750    3.619031029899    5.437172998971
 na>    1.000000000000     3.131836352550   -1.521407564485    5.437881058244
 na>    1.000000000000     3.032930527372    1.823930793162    5.495517083096
 na>    1.000000000000     3.076486816200    1.880198637181   -5.574904688825
 y>     3.000000000000    -2.966783394600   -5.160194478640    3.541273488613
 y>     3.000000000000    -5.990436598340    0.077223391515   -3.571974938706
 y>     3.000000000000    -5.995334522170    0.064545621600    3.552970627809
 y>     3.000000000000    -2.963709629066   -5.089549595429   -0.007505428298
 y>     3.000000000000     0.051218650999   -3.417905851849   -1.776336621457
 y>     3.000000000000    -2.996338510160    1.807209489822    5.291029564949
 y>     3.000000000000    -2.977665474977    1.817487369832   -1.777143809029
 y>     3.000000000000     0.069307304399   -3.389000090115    5.290208216191
 y>     3.000000000000     3.072635176974   -5.181828655114    3.536543652667
 y>     3.000000000000     0.021334790413    0.094965246924   -3.582411732396
 y>     3.000000000000     0.026551079810    0.097541355053    3.527069819590
 y>     3.000000000000     3.081924216514   -5.123096876113    0.003582779923
 y>     3.000000000000     3.042860219791    1.816025759712   -1.859505263707
 y>     3.000000000000     3.063326589178    1.807281083487    1.843361512274
 y>     3.000000000000    -2.963104823677   -5.176898325394   -3.580613261841
 y>     3.000000000000    -5.989407364360    0.025273006708   -0.011031563479
 f>    -0.999866027919    -5.044085023025   -2.051517651206    0.136131475897   NewECP "SDD" end
 f>    -0.999929646478    -4.997320938612   -2.048592091175    3.338796858802   NewECP "SDD" end
 f>    -0.999937704878    -4.971807286311   -2.055538839272   -3.656842923212   NewECP "SDD" end
 f>    -0.999998266478    -1.446439206195   -5.254674339321   -1.769171061611   NewECP "SDD" end
 f>    -0.999966330782    -1.541751994445    5.324484613547   -1.783077345740   NewECP "SDD" end
 f>    -0.999673930815     0.722885314723    5.504178375816    0.144316641097   NewECP "SDD" end
 f>    -0.999645101918     0.730143690463    5.519405288202   -3.712298125502   NewECP "SDD" end
 f>    -0.999662701635     0.726844167569    5.505766164915    3.288680423434   NewECP "SDD" end
 y>     2.999823058018    -3.002824854802    5.350599725562    0.003582779923
 f>    -0.999946800387     5.329685707741    1.439336365092   -1.779055569067   NewECP "SDD" end
 f>    -0.999876770699     5.360462610831    1.486925853619    1.762033824135   NewECP "SDD" end
 y>     2.999958845034     6.086591635640    0.025273006708   -0.011031563479
 y>     2.999561411107     3.027540299618    5.384147006247   -0.007505428298
*
```

Full Calculation of Merged Fragments:

```
# CASSCF+NEVPT2+QDPT calculation of 12-NaYF4: 5.0 % Er3+ (Er-F9-Na18Y19F61) with 35 quartet and 112 doublet roots

% Pal NProcs 24 end                  # number of cores for parallel processing
% MaxCore 3000                       # amount of RAM per core

% SCF
  Guess MORead                       # restart from an earlier calculation
  MOInp "merged.gbw"       # merged ligand and metal orbitals
end

% PointCharges "pointcharges.pc"     # file that contains all point charges
% Method
  DoEQ true                          # include interactions between point charges
  FrozenCore FC_None       # no frozencore approximation, calculates correlation effects for electrons on low-lying orbitals
end

% CASSCF
  NEl 11                             # number of electrons
  NOrb 7                             # number of orbitals
  Mult 4,2                 # included multiplicities
  NRoots 35,112                      # number of multiplets per multiplicity
  BWeight = 0.238095, 0.761905       # weight per mutliplicity block
  MaxIter 500              # maximum number of iterations to achieve convergence criteria
  ETol 1e-8                # energy convergence setting (default is 1e-7)
  GTol 1e-5                # gradient convergence setting
  ActConstraints 0         # requests natural orbital construction
  trafostep RI             # utilize the RI-MO algorithm for faster integral transformation
  ActOrbs fOrbs                      # requests AILFT analysis
  PTMethod SC_NEVPT2       # strongly contracted MR approach, provides a DEC corrected total energy
  PTSettings               # settings for the chosen perturbation theory method
   D3Tpre 1e-15                      # truncation of the third-order reduced density matrix (tight setting)
   D4Tpre 1e-15                      # fourth-order rdm, avoids intruder states (extremely tight setting)
  end
  DoHigherMoments true     # request the multipole expansion up to 2nd order (magnetic dipole and electric quadrupole contributions), introduces origin-dependence
  rel                                # enter into relativistic calculation
   DoSOC true              # spin-orbit coupling via QDPT
```

```
   NInitStates 364          # number of inital SOC states to compute the transition moments from
   Temperature 1000000      # temperature for calculating the Boltzmann distribution
  end
end

% Rel                                   # include relativistic effects
  Method DKH                # (relativistic) Hamiltonian operator
  Order 2                   # second-order DKH Hamiltonian
  PictureChange 2           # include the second-order DKH transformation of the SOC operator and the first order of the Zeeman operator
  FiniteNuc true                        # account for the spatial extent of the nuclei (opposed to the point charges used for the elementary particles)
end

! AutoAux                   # automatic construction of a auxiliary basis set for Coulomb-, exchange-, and correlation-fitting
% Basis
   Basis "DKH-DEF2-TZVPP"   # general basis set
  NewGTO
   Er "SARC2-DKH-QZVP" end
  NewECP                    # ECP per element
   Na "SDD" end
  NewECP                    # ECP per element
   Y "SDD" end
end

* xyz -6 4                  # keyword, total charge, multiplicity, (file name)
  f    -2.299492273536    0.308637372811   -0.153181543199
  f    -0.818879087031   -1.217957239365   -1.788458596217
  f    -0.749118336035   -1.180309559734    1.766083923179
  f     0.957298398847   -2.089301717118   -0.156636872453
  f    -0.624558199710    1.385730558004    1.705105858557
  f    -0.633420712704    1.448685779723   -1.731034989147
  f     1.620272740359   -0.048917365162    1.708122191061
  f     1.585444746148    0.027856445483   -1.732012110945
  f     1.501069922024    1.966620981619    0.015364886232
   f>   -1.000000000000   -3.616969752303   -3.742219477545    1.756964119738   NewECP "SDD" end
   f>   -1.000000000000   -1.483281727066   -5.213510369862    1.711011072897   NewECP "SDD" end
   f>   -1.000000000000   -4.554457480747    0.073984742263    1.759442327195   NewECP "SDD" end
   f>   -1.000000000000   -4.471358951069   -0.056232986376   -1.780330075759   NewECP "SDD" end
   f>   -1.000000000000   -4.503300747424    0.037250296028   -5.290944597836   NewECP "SDD" end
   f>   -1.000000000000   -4.431057887467   -0.071949672423    5.268853148507   NewECP "SDD" end
   f>   -1.000000000000   -4.701662530124    1.976313126456   -0.165728353523   NewECP "SDD" end
   f>   -1.000000000000   -1.653017848711   -3.247327998787   -3.360760857462   NewECP "SDD" end
   f>   -1.000000000000   -4.676890702508    1.985495062150   -3.357234722281   NewECP "SDD" end
   f>   -1.000000000000   -4.663113037556    1.969747961160    3.690886413075   NewECP "SDD" end
   f>   -1.000000000000   -1.617248029872   -3.254848963274   -0.164666264613   NewECP "SDD" end
   f>   -1.000000000000   -1.638595671890   -3.230301453121    3.641605487649   NewECP "SDD" end
   f>   -1.000000000000    0.749223038664   -5.006143580048   -0.210704278567   NewECP "SDD" end
   f>   -1.000000000000   -2.310979368770    0.280778569443   -3.360732535091   NewECP "SDD" end
   f>   -1.000000000000   -2.295118581786    0.275918003966    3.627500946924   NewECP "SDD" end
   f>   -1.000000000000    0.772107127869   -4.956249997758   -3.358395939489   NewECP "SDD" end
   f>   -1.000000000000    0.766493418574   -4.915732239621    3.689555261641   NewECP "SDD" end
   f>   -1.000000000000   -0.817733798160   -1.228162188904    5.269065566289   NewECP "SDD" end
   f>   -1.000000000000   -3.816450895798    4.008431336318   -1.781094779774   NewECP "SDD" end
   f>   -1.000000000000   -0.798563277331   -1.172839559706   -5.290052443151   NewECP "SDD" end
   f>   -1.000000000000   -3.833265813567    4.052240491136    1.759286554155   NewECP "SDD" end
   f>   -1.000000000000   -2.058737219729    3.188307177482    3.683012793955   NewECP "SDD" end
   f>   -1.000000000000    1.004145483932   -2.038644391723   -3.369781532605   NewECP "SDD" end
   f>   -1.000000000000   -1.996971619225    3.185009744230   -3.361568045034   NewECP "SDD" end
   f>   -1.000000000000    1.033751495854   -2.015588605525    3.636394171397   NewECP "SDD" end
   f>   -1.000000000000   -1.994431917776    3.209551431044   -0.157231642243   NewECP "SDD" end
   f>   -1.000000000000   -0.711936165766    1.396138800890   -5.235333622506   NewECP "SDD" end
   f>   -1.000000000000    2.340946409013   -3.876635334905    1.764257130254   NewECP "SDD" end
   f>   -1.000000000000    2.378521906020   -3.840575335991   -5.288508873935   NewECP "SDD" end
   f>   -1.000000000000   -0.688728740098    1.483251201001    5.209815166294   NewECP "SDD" end
   f>   -1.000000000000    2.407802943759   -3.744709456566   -1.783884533311   NewECP "SDD" end
   f>   -1.000000000000    2.433436540495   -3.706981847047    5.268442474128   NewECP "SDD" end
   f>   -1.000000000000    1.517090797101   -0.036550616726   -5.287517590953   NewECP "SDD" end
   f>   -1.000000000000    1.479107813772    0.069602739607    5.268428312943   NewECP "SDD" end
   f>   -1.000000000000   -1.475470844758    5.167587296065    1.767330107500   NewECP "SDD" end
   f>   -1.000000000000    4.437201106207   -3.274037525842   -3.712864572920   NewECP "SDD" end
   f>   -1.000000000000    1.392532127605    1.976574173895   -3.415366388624   NewECP "SDD" end
   f>   -1.000000000000    4.429250373208   -3.269262433015    3.341855674863   NewECP "SDD" end
   f>   -1.000000000000    1.386101387971    1.972220299282    3.336516907942   NewECP "SDD" end
   f>   -1.000000000000    4.460341376552   -3.303762316061    0.190382977422   NewECP "SDD" end
   f>   -1.000000000000    3.725996229721    0.283955256882   -3.419345681740   NewECP "SDD" end
   f>   -1.000000000000    3.724910305357    0.266818222262    3.393359906408   NewECP "SDD" end
   f>   -1.000000000000    3.738996653948    0.461249658737    0.012603455066   NewECP "SDD" end
   f>   -1.000000000000    2.190011989058    3.967955283430   -1.730865054922   NewECP "SDD" end
   f>   -1.000000000000    5.184453948238   -1.186394384778    1.754613362950   NewECP "SDD" end
   f>   -1.000000000000    2.243909262942    3.966018355847    1.706819361998   NewECP "SDD" end
   f>   -1.000000000000    5.250111275293   -1.170458331321   -1.778135092012   NewECP "SDD" end
   f>   -1.000000000000    4.011660262190    3.140229669000   -0.039453062712   NewECP "SDD" end
   f>   -1.000000000000    4.035485396296    3.187065114589    3.391009149620   NewECP "SDD" end
   f>   -1.000000000000    4.046317139562    3.192080455337   -3.362587650387   NewECP "SDD" end
   f>   -1.000000000000   -3.722048656733   -3.924665774858   -1.786773415147   NewECP "SDD" end
  na>     1.000000000000    -3.062343860357   -1.804378518666   -2.076964427742
  na>     1.000000000000    -3.069555754538   -1.812246470978    5.321858465711
  na>     1.000000000000    -2.947545973451   -1.606851014237   -5.287503429767
  na>     1.000000000000    -2.904136620751   -1.519840691063    1.882248127567
  na>     1.000000000000     0.042149882197   -3.410402411246    1.823422563136
  na>     1.000000000000    -2.991025551500    1.833991004062   -5.400453045054
  na>     1.000000000000    -2.999516923814    1.828112942700    1.766310502146
  na>     1.000000000000     0.079003550537   -3.354858013524   -5.343411789992
  na>     1.000000000000     2.996384747963   -1.763228909509    2.170187511673
  na>     1.000000000000    -0.015930357109    3.523022365965    1.652086380167
```

```
na>   1.000000000000    3.060192958885  -1.656433202894  -5.519987611583
na>   1.000000000000   -0.041059624125   3.674240626765  -1.674758438101
na>   1.000000000000    0.105476993763   3.435442983838  -5.458712162066
na>   1.000000000000    3.064271237285  -1.662608618084  -2.306970402101
na>   1.000000000000    0.036136765750   3.619031029899   5.437172998971
na>   1.000000000000    3.131836352550  -1.521407564485   5.437881058244
na>   1.000000000000    3.032930527372   1.823930793162   5.495517083096
na>   1.000000000000    3.076486816200   1.880198637181  -5.574904688825
y>    3.000000000000   -2.966783394600  -5.160194478640   3.541273488613
y>    3.000000000000   -5.990436598340   0.077223391515  -3.571974938706
y>    3.000000000000   -5.995334522170   0.064545621600   3.552970627809
y>    3.000000000000   -2.963709629066  -5.089549595429  -0.007505428298
y>    3.000000000000    0.051218650999  -3.417905851849  -1.776336621457
y>    3.000000000000   -2.996338510160   1.807209489822   5.291029564949
y>    3.000000000000   -2.977665474977   1.817487369832  -1.777143809029
y>    3.000000000000    0.069307304399  -3.389000090115   5.290208216191
y>    3.000000000000    3.072635176974  -5.181828655114   3.536543652667
y>    3.000000000000    0.021334790413   0.094965246924  -3.582411732396
y>    3.000000000000    0.026551079810   0.097541355053   3.527069819590
y>    3.000000000000    3.081924216514  -5.123096876113   0.003582779923
y>    3.000000000000    3.042860219791   1.816025759712  -1.859505263707
y>    3.000000000000    3.063326589178   1.807281083487   1.843361512274
y>    3.000000000000   -2.963104823677  -5.176898325394  -3.580613261841
y>    3.000000000000   -5.989407364360   0.025273006708  -0.011031563479
f>   -0.999866027919   -5.044085023025  -2.051517651206   0.136131475897  NewECP "SDD" end
f>   -0.999929646478   -4.997320938612  -2.048592091175   3.338796858802  NewECP "SDD" end
f>   -0.999937704878   -4.971807286311  -2.055538839272  -3.656842923212  NewECP "SDD" end
f>   -0.999998266478   -1.446439206195  -5.254674339321  -1.769171061611  NewECP "SDD" end
f>   -0.999966330782   -1.541751994445   5.324484613547  -1.783077345740  NewECP "SDD" end
f>   -0.999673930815    0.722885314723   5.504178375816   0.144316641097  NewECP "SDD" end
f>   -0.999645101918    0.730143690463   5.519405288202  -3.712298125502  NewECP "SDD" end
f>   -0.999662701635    0.726844167569   5.505766164915   3.288680423434  NewECP "SDD" end
y>    2.999823058018   -3.002824854802   5.350599725562   0.003582779923
f>   -0.999946800387    5.329685707741   1.439336365092  -1.779055569067  NewECP "SDD" end
f>   -0.999876770699    5.360462610831   1.486925853619   1.762033824135  NewECP "SDD" end
y>    2.999958845034    6.086591635640   0.025273006708  -0.011031563479
y>    2.999561411107    3.027540299618   5.384147006247  -0.007505428298
Er    0.000000000000    0.000000000000   0.000000000000
*
```

6 References

(1) Dulick, M.; Faulkner, G. E.; Cockroft, N. J.; Nguyen, D. C. Spectroscopy and Dynamics of Upconversion in Tm^{3+}: $YLiF_4$. *J. Lumin.* **1991**, *48-49*, 517−521. DOI: https://doi.org/10.1016/0022-2313(91)90183-V.

(2) Xiao, Y.; Kuang, X.; Yeung, Y.; Ju, M. Investigation of the Structure and Luminescence Mechanism of Tm^{3+}-Doped $LiYF_4$: New Theoretical Perspectives. *Inorg. Chem.* **2020**, *59* (2), 1211−1217. DOI: https://doi.org/10.1021/acs.inorgchem.9b02935.

(3) Sugiyama, A.; Katsurayama, M.; Anzai, Y.; Tsuboi, T. Spectroscopic Properties of Yb Doped YLF Grown by a Vertical Bridgman Method. *J. Alloys Compd.* **2006**, *408-412*, 780−783. DOI: https://doi.org/10.1016/j.jallcom.2005.01.094.

(4) Rinkel, T.; Nordmann, J.; Raj, A. N.; Haase, M. Ostwald-Ripening and Particle Size Focussing of Sub-10 nm $NaYF_4$ Upconversion Nanocrystals. *Nanoscale* **2014**, *6* (23), 14523−14530. DOI: https://doi.org/10.1039/C4NR03833A.

(5) Liu, N.; Gobeil, N.; Evers, P.; Gessner, I.; Rodrigues, E. M.; Hemmer, E. Water Dispersible Ligand-Free Rare Earth Fluoride Nanoparticles: Water Transfer Versus $NaREF_4$-to-REF_3 Phase Transformation. *Dalton Trans.* **2020**, *49* (45), 16204−16216. DOI: https://doi.org/10.1039/D0DT01080D.
